# Atom-Economic Synthesis of 4-Pyrones from Diynones and Water

**DOI:** 10.3390/molecules22010109

**Published:** 2017-01-10

**Authors:** Yan-Li Xu, Qing-Hu Teng, Wei Tong, Heng-Shan Wang, Ying-Ming Pan, Xian-Li Ma

**Affiliations:** 1 College of Pharmacy, Guilin Medical University, Guilin 541004, China; yanlixu@163.com; 2 State Key Laboratory for Chemistry and Molecular Engineering of Medicinal Resources, School of Chemistry and Pharmaceutical Sciences of Guangxi Normal University, Guilin 541004, China; qinghuteng@163.com (Q.-H.T.); m18778391736@163.com (W.T.); whengshan@163.com (H.-S.W.)

**Keywords:** 4-pyrones, diynones, water, transition-metal-free

## Abstract

Transition-metal-free synthesis of 4-pyrones via TfOH-promoted nucleophilic addition/cyclization of diynones and water has been developed. This transformation is simple, atom economical and environmentally benign, providing rapid and efficient access to substituted 4-pyrones.

## 1. Introduction

Water (H_2_O) is inexpensive, safe, and environmentally friendly [1]. It is the most economic and eco-friendly solvent available in Nature and therefore highly desirable for chemical reactions [2]. Generally, water offers several “green chemistry” benefits as a solvent in organic transformations, including high efficiency, lower cost, ease of process, green and environmental protection [3,4]. Recently, there are many reports of clean transformations in water medium [5,6,7,8,9,10,11,12,13,14,15,16,17,18,19], such as coupling reactions [20,21,22,23,24,25,26,27,28,29,30], cyclizations [31,32,33,34], Michael additions [35,36,37,38,39], and condensations [40,41]. Additionally, H_2_O also participates in organic reactions as a nucleophile [42,43] to provide various kinds of functional compounds such as imidazo[1,2-*a*]pyridines [44], amino acid salts [45], α-amino ketones [46], and 1,3-oxazinan-2-ones [47]. Thus, the studies of organic reactions in aqueous solvents or H_2_O-participating reactions are attractive in synthetic chemistry.

**Figure 1:**
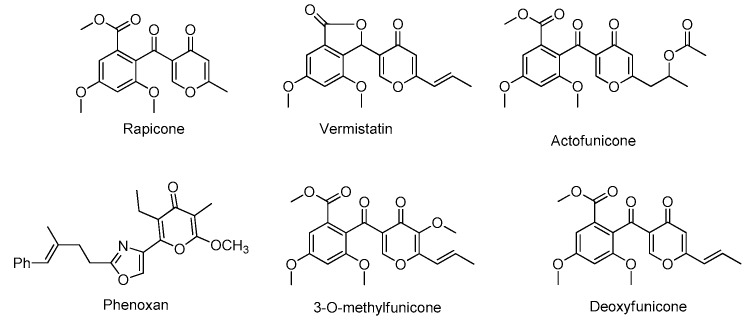
4-Pyrones disclosed as biologically active organic molecules.

4-Pyrones are heterocycles with multiple biological activities [48,49,50], which are widely found in biologically active natural products and functional chemicals [51,52,53,54,55,56,57,58,59]. Particularly, phenoxans, funicones and rapicones possess potent anti-HIV activity (Figure 1) [60,61,62]. In general, 4-pyrones are prepared via the well-known condensation cyclization reaction of carbonyl compounds with polystep reactions [63,64,65,66,67]. Additionally, a transformation of isoxazoles to substituted pyran-4-ones in the presence of Mo(CO)_6_ and HCO_2_H in a two-step procedure was established [68]. Although these reported methods have made significant contributions to the applications of 4-pyrones in pharmacology and food manufacture [69], the development of efficient and practical synthetic methods for 4-pyrones from easily accessible starting materials is still highly desirable. Continuing our interest in the conversion of alkynes to heterocycles [70,71,72,73,74,75,76,77], herein, we would like to describe an efficient, transition-metal-free synthesis of 4-pyrones through TfOH-promoted cyclization of diynones. Water acts as both the substrate and solvent, obviating the need for an organic co-solvent. Overall, the reaction is atom-economical and environmentally benign.

## 2. Results and Discussion

1,5-Diphenylpenta-1,4-diyn-3-one (**1a**) was chosen as model substrate to identify the optimal conditions for this reaction (Table 1). Originally, the reaction was carried out in the presence of 1 equiv. TfOH for 24 h to afford the desired product **2a** in 70% yield (Table 1, entry 1). When other acid catalysts such as CH_3_COOH, PTSA, HCl, H_3_PO_4_ and PhCOOH were screened, the yield of **2a** decreased (Table 1, entries 2–6). Further experiments demonstrated that decreasing the amount of TfOH was detrimental to the yield of **2a** (Table 1, entries 7 and 8), and no obvious improvement of yield was noted by using 2 equiv. of TfOH (Table 1, entry 9). Poor yield of **2a** was obtained when the reaction was performed at 80 °C, while not much change was noted between 100 °C and 130 °C (Table 1, entries 10 and 11). In addition, an 83% yield was achieved when the reaction time was extended to 36 h (Table 1, entry 12). Thus, the best conditions for this transformation involved 1 equiv. of TfOH in H_2_O at 100 °C for 36 h.

**Scheme 1:**
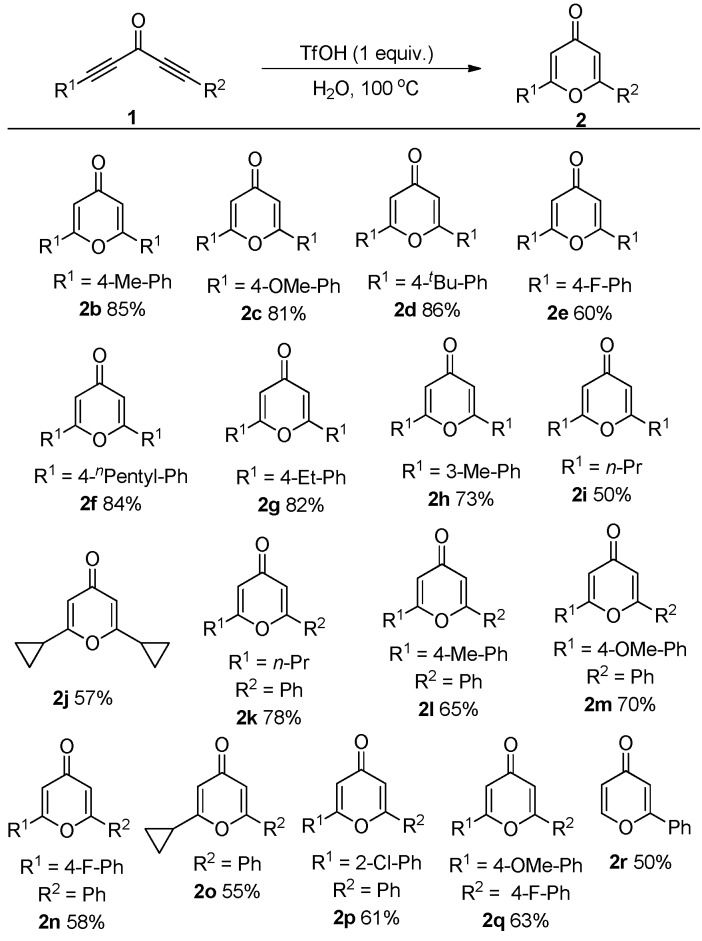
Synthesis of 4-pyrone derivatives ^a,b^. ^a^ Reaction conditions: **1** (0.5 mmol), TfOH (1 equiv.), H_2_O (1 mL), at 100 °C, 36 h; ^b^ Isolated yields.

**Scheme 2:**
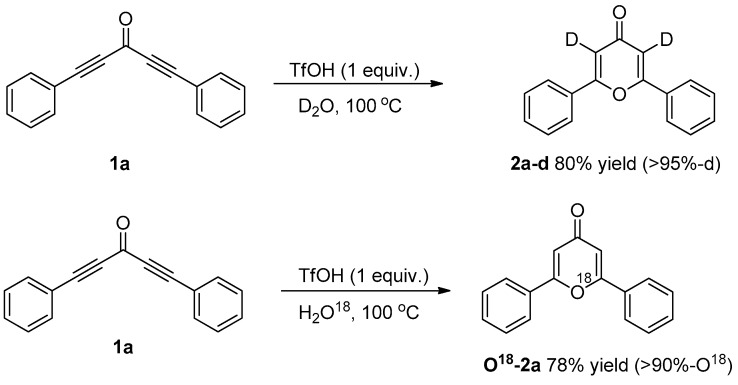
Control experiments.

Under the optimized reaction conditions, the one-pot reaction worked well using all kinds of diynones, as shown in Scheme 1. Firstly, various symmetric diynones were identified as suitable substrates for the reaction and provided the desired products in moderate to good yields (Scheme 1, **2b**–**2j**). Aryl groups with electron-donating groups (EDG) gave satisfactory yields (Scheme 1, **2b**–**2d** and **2f**–**2h**), whereas aryl groups with electron-withdrawing groups (EWG) afforded slightly lower yields (Scheme 1, **2e**). Gratifyingly, aliphatic diynones worked smoothly to generate the corresponding cyclization products **2i** and **2j** in 50% and 57%, respectively (Scheme 1, **2i** and **2j**). After exploring the reaction substrate scope of symmetric diynones, we next examined asymmetric diynones substrates. To our delight, the corresponding 4-pyrones products were obtained in moderate to good yields under the standard conditions (Scheme 1, **2k**–**2r**). The desired products **2k**–**2q** were obtained in 55%–78% yields when asymmetric diynones substrates **1k**–**1q** (R^2^ = Ph, R^1^ = aryl- or alkyl-) were subjected to this reaction. Obviously, aryl groups with electron-donating groups gave higher yields than diynones featuring electron-withdrawing groups on the phenyl ring (Scheme 1, **2l** and **2m** vs. **2n** and **2p**). Notably, diynone **1p**, which possess an electron-withdrawing group at the *ortho-*position of the phenyl ring (R^1^ = 2-Cl-Ph, R^2^ = Ph) reacted readily to afford **2p** in 61% yield (Scheme 1, **2p**). Furthermore, diynone **1q**, which bear both a EDG-incorporated aryl ring and a EWG-incorporated aryl ring (R^1^ = 4-OMe-Ph, R^2^ = 4-F-Ph) also participated well in the reaction and offered **2q** in 63% yield (Scheme 1, **2q**). Finally, diynone **1r** also worked smoothly to give **2r** in 50% yield (Scheme 1, **2r**).

To better understand the reaction mechanism, we carried out control experiments as outlined in Scheme 2. Deuterium-labeled D_2_O was used in the reaction with diynone **1a** to give the deuterium-labeled product **2a**-**d** in 80% yield, where over 95% of deuterium was incorporated into the cyclization product.

This result demonstrated that H_2_O was introduced into the 4-pyrones. Moreover, an O^18^-labeled experiment further showed that H_2_O reacted with diynones to form 4-pyrones.

**Scheme 3:**
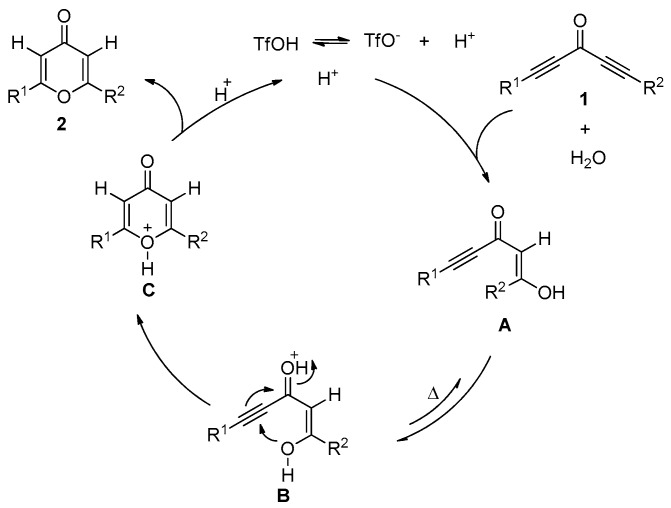
Proposed mechanism.

On the basis of the above results and existing literature [78], a plausible mechanistic description of the nucleophilic addition and cyclization reaction is shown in Scheme 3. First, the carbonyl of the diynone substrate was activated by TfOH, followed by nucleophilic addition of H_2_O to the carbon−carbon triple bond of diynone and keto–enol tautomerization [79,80] to form intermediate **A**. Then intermediate **A** was converted to **B** through protonation and C–C bond rotation, which was promoted by elevated temperature. Subsequently, an intramolecular nucleophilic attack of the oxhydryl group to the carbon−carbon triple bond of **B** lead to a cyclization intermediate **C**. Finally, deprotonation of **C** gave the desired 4-pyrone **2**.

**Scheme 4:**
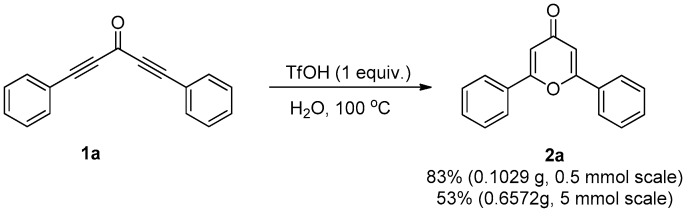
Gram-scale synthesis.

The treatment of 1,5-diphenylpenta-1,4-diyn-3-one **1a** in H_2_O at 100 °C for 36 h in the presence of TfOH afforded the corresponding cyclization product **2a** in 83% yield. The preparation of this compound **2a** on gram-scale afforded 53% of the isolated product (Scheme 4).

## 3. Materials and Methods

### 3.1. General Information

All manipulations were performed under an air atmosphere unless otherwise stated. Column chromatography was performed on silica gel (300–400 mesh). NMR spectra were obtained using an Avance 500 spectrometer (^1^H at 500 MHz and ^13^C at 125 MHz) or an Avance 400 spectrometer (^1^H at 400 MHz and ^13^C at 100 MHz) (Bruker Corporation, Karlsruhe, Germany). IR spectra were recorded on a Nicolet ESP 360 FT-IR spectrometer (Nicolet, Madison, WI, USA) and only major peaks are reported in cm^−1^. High resolution mass spectra (HRMS) were recorded on an Exactive Mass Spectrometer (Thermo Fisher Scientific, Waltham, MA, USA) equipped with ESI or APCI ionization sources. Unless stated otherwise, commercial reagents were used without further purification. All reagents were weighed and handled at room temperature. Compounds **1a**–**1r** were prepared by the reported methods [78,81]. The NMR spectra and HRMS spectra of the products can be found in the Supplementary Materials.

### 3.2. General Procedure for the Synthesis of Compound ***2***

**Scheme 5:**
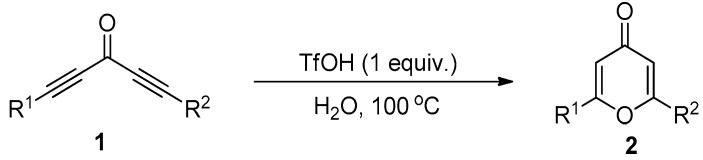
Synthesis of Compounds **2**.

The reaction mixture of **1** (0.5 mmol), TfOH (1 equiv.) and H_2_O (1 mL) in a 15 mL test tube was stirred at 100 °C for 36 h, and monitored periodically by TLC. Upon completion, the reaction mixture was diluted with water (5 mL) and extracted with ethyl acetate (3 × 5 mL). The combined organic layers were washed with water and brine, dried over MgSO_4_ and filtered. The solvent was removed under vacuum. The residue was purified by flash column chromatography (petroleum ether and ethyl acetate, *v*/*v* = 5:1 to 2:1) to afford 4-pyrones **2** (Scheme 5).

*2,6-Diphenyl-4H-pyran-4-one* (**2a**) [82]. The general procedure was used with 1,5-diphenylpenta-1,4-diyn-3-one (115.04 mg, 0.5 mmol, 1 equiv.) and water (1 mL). The crude obtained was purified by column chromatography (petroleum ether/ethyl acetate = 5:1 to 2:1) to afford the product as a yellow solid (102.90 mg, 83%); m.p. 135.3–136.2 °C (lit: 139–140 °C); ^1^H-NMR (500 MHz, CDCl_3_) δ 7.89–7.82 (m, 4H), 7.55–7.50 (m, 6H), 6.81 (s, 2H) ppm; ^13^C-NMR (125 MHz, CDCl_3_) δ 180.2, 163.3, 131.4, 131.4, 129.1, 125.9, 111.4 ppm; IR (KBr): 3060, 2925, 1647, 1614, 1604, 1493, 1450, 1392, 943, 770, 683 cm^−1^; HRMS (*m*/*z*) (APCI): calcd. for C_17_H_13_O_2_ 249.0917 [M + H^+^]; found 249.0906.

*2,6-Di-p-tolyl-4H-pyran-4-one* (**2b**) [78]. The general procedure was used with 1,5-di-*p*-tolylpenta-1,4-diyn-3-one (129.05 mg, 0.5 mmol, 1 equiv.) and water (1 mL). The crude obtained was purified by column chromatography (petroleum ether/ethyl acetate = 5:1 to 2:1) to afford the product as a yellow solid (117.35 mg, 85%); m.p. 180.5–183.1 °C (lit: 178 °C); ^1^H-NMR (500 MHz, CDCl_3_) δ 7.74 (d, *J* = 8.2 Hz, 42H), 7.32 (d, *J* = 8.0 Hz, 4H), 6.76 (s, 2H), 2.43 (s, 6H) ppm; ^13^C-NMR (125 MHz, CDCl_3_) δ 180.4, 163.4, 141.9, 129.8, 128.7, 125.8, 110.7, 21.5 ppm; IR (KBr): 3066, 1646, 1605, 1507, 1413, 1383, 942, 819, 478 cm^−1^; HRMS (*m*/*z*) (APCI): calcd. for C_19_H_17_O_2_ 277.1230 [M + H^+^]; found 277.1219.

*2,6-Bis(4-methoxyphenyl)-4H-pyran-4-one* (**2c**) [82]. The general procedure was used with 1,5-bis(4-methoxyphenyl)penta-1,4-diyn-3-one (145.05 mg, 0.5 mmol, 1 equiv.) and water (1 mL). The crude obtained was purified by column chromatography (petroleum ether/ethyl acetate = 5:1 to 2:1) to afford the product as a yellow solid (124.78 mg, 81%); m.p. 190–193.8 °C (lit: 189–191 °C); ^1^H-NMR (500 MHz, CDCl_3_) δ 7.79 (d, *J* = 8.9 Hz, 4H), 7.02 (d, *J* = 8.9 Hz, 4H), 6.70 (s, 2H), 3.88 (s, 6H) ppm; ^13^C-NMR (125 MHz, CDCl_3_) δ 163.2, 162.1, 134.4, 127.5, 123.9, 114.5, 109.7, 55.5 ppm; IR (KBr): 2983, 2875, 2765, 1651, 1607, 1507, 1387, 1262, 1226, 1177, 1020, 829 cm^−1^; HRMS (*m*/*z*) (APCI): calcd. for C_19_H_17_O_4_ 309.1129 [M + H^+^]; found 309.1115.

*2,6-Bis(4-(tert-butyl)phenyl)-4H-pyran-4-one* (**2d**) [82]. The general procedure was used with 1,5-bis(4-(*tert*-butyl)phenyl)penta-1,4-diyn-3-one (171.10 mg, 0.5 mmol, 1 equiv.) and water (1 mL). The crude obtained was purified by column chromatography (petroleum ether/ethyl acetate = 5:1 to 2:1) to afford the product as a yellow solid (154.89 mg, 86%); m.p. 192.5–193.1 °C (lit: 192–194 °C); ^1^H-NMR (500 MHz, CDCl_3_) δ 7.79 (d, *J* = 7.2 Hz, 4H), 7.54 (d, *J* = 7.4 Hz, 4H), 6.81 (s, 2H), 1.36 (s, 18H) ppm; ^13^C-NMR (125 MHz, CDCl_3_) δ 180.5, 163.5, 155.0, 128.6, 126.0, 125.7, 34.9, 31.0 ppm; IR (KBr): 3064, 3003, 2998, 2970, 2868, 1715, 1667, 1650, 1450, 1340, 1250, 910 cm^−1^; HRMS (*m*/*z*) (APCI): calcd. for C_25_H_29_O_2_ 361.2169 [M + H^+^]; found 361.2153.

*2,6-Bis(4-fluorophenyl)-4H-pyran-4-one* (**2e**) [82]. The general procedure was used with 1,5-bis(4-fluorophenyl)penta-1,4-diyn-3-one (133.12 mg, 0.5 mmol, 1 equiv.) and water (1 mL). The crude obtained was purified by column chromatography (petroleum ether/ethyl acetate = 5:1 to 2:1) to afford the product as a white solid (85.22 mg, 60%); m.p. 160–161.3 °C (lit: 167–170 °C); ^1^H-NMR (500 MHz, CDCl_3_) δ 7.84 (dd, *J* = 8.5, 5.2 Hz, 4H), 7.22 (t, *J* = 8.4 Hz, 4H), 6.75 (s, 2H) ppm; ^13^C-NMR (125 MHz, CDCl_3_) δ 179.9, 164.6 (d, *J* = 253.4 Hz), 162.5, 128.1 (d, *J* = 8.8 Hz), 127.6 (d, *J* = 3.3 Hz), 116.5(d, *J* = 22.2 Hz), 111.3 ppm; IR (KBr): 3059, 2924, 1662, 1599, 1504, 1417, 1380, 1241, 1223, 1160, 837 cm^−1^; HRMS (*m*/*z*) (APCI): calcd. for C_17_H_11_F_2_O_2_ 285.0729 [M + H^+^]; found 285.0716.

*2,6-Bis(4-pentylphenyl)-4H-pyran-4-one* (**2f**). The general procedure was used with 1,5-bis(4-pentylphenyl)penta-1,4-diyn-3-one (185.11 mg, 0.5 mmol, 1 equiv.) and water (1 mL). The crude obtained was purified by column chromatography (petroleum ether/ethyl acetate = 5:1 to 2:1) to afford the product as a yellow solid (163.06 mg, 84%); m.p. 66.7–67.9 °C; ^1^H-NMR (500 MHz, CDCl_3_) δ 7.76 (d, *J* = 8.1 Hz, 4H), 7.32 (d, *J* = 8.1 Hz, 4H), 6.77 (s, 2H), 2.70–2.66 (m, 4H), 1.69–1.61 (m, 4H), 1.36–1.33 (m, 8H), 0.90 (t, *J* = 6.9 Hz, 6H) ppm; ^13^C-NMR (125 MHz, CDCl_3_) δ 180.5, 163.5, 146.9, 129.2, 128.9, 125.9, 110.7, 35.8, 31.4, 30.8, 22.5, 13.9 ppm; IR (KBr): 3032, 2956, 2929, 2857, 1717, 1649, 1609, 1419, 1380, 1186, 944, 849, 649 cm^−1^; HRMS (*m*/*z*) (APCI): calcd. for C_27_H_33_O_2_ 389.2482 [M + H^+^]; found 389.2466.

*2,6-Bis(4-ethylphenyl)-4H-pyran-4-one* (**2g**) [78]. The general procedure was used with 1,5-bis(4-ethylphenyl)penta-1,4-diyn-3-one (143.07 mg, 0.5 mmol, 1 equiv.) and water (1 mL). The crude obtained was purified by column chromatography (petroleum ether/ethyl acetate = 5:1 to 2:1) to afford the product as a brown solid (124.70 mg, 82%); m.p. 119.5–121.5 °C; ^1^H-NMR (500 MHz, CDCl_3_) δ 7.77 (d, *J* = 8.1 Hz, 4H), 7.34 (d, *J* = 8.1 Hz, 4H), 6.77 (s, 2H), 2.72 (q, *J* = 7.6 Hz, 4H), 1.28 (t, *J* = 7.6 Hz, 6H) ppm; ^13^C-NMR (125 MHz, CDCl_3_) δ 180.4, 163.5, 148.2, 128.9, 128.6, 125.9, 110.6, 28.8, 15.2 ppm; IR (KBr): 3070, 2965, 2875, 1647, 1610, 1510, 1451, 1420, 1383, 1187, 1014, 945, 837, 643 cm^−1^; HRMS (*m*/*z*) (APCI): calcd. for C_21_H_21_O_2_ 305.1543 [M + H^+^]; found 305.1532.

*2,6-Di-m-tolyl-4H-pyran-4-one* (**2h**) [78]. The general procedure was used with 1,5-di-*m*-tolylpenta-1,4-diyn-3-one (129.05 mg, 0.5 mmol, 1 equiv.) and water (1 mL). The crude obtained was purified by column chromatography (petroleum ether/ethyl acetate = 5:1 to 2:1) to afford the product as a light yellow solid (100.78 mg, 73%); m.p. 73.5–75.5 °C; ^1^H-NMR (500 MHz, CDCl_3_) δ 7.66–7.62 (t, *J* = 7.6 Hz, 4H), 7.41 (t, *J* = 7.6 Hz, 2H), 7.34 (d, *J* = 7.6 Hz, 2H), 6.78 (s, 2H), 2.45 (s, 6H) ppm; ^13^C-NMR (125 MHz, CDCl_3_) δ 163.6, 138.9, 132.1, 131.4, 129.0, 126.5, 123.1, 111.3, 21.5 ppm; IR (KBr): 3063, 2923, 1646, 1611, 1485, 1384, 1260, 1075, 929, 784, 694, 435 cm^−1^; HRMS (*m*/*z*) (APCI): calcd. for C_19_H_17_O_2_ 277.1230 [M + H^+^]; found 277.1219.

*2,6-Dipropyl-4H-pyran-4-one* (**2i**) [78]. The general procedure was used with undeca-4,7-diyn-6-one (81.05 mg, 0.5 mmol, 1 equiv.) and water (1 mL). The crude obtained was purified by column chromatography (petroleum ether/ethyl acetate = 5:1 to 2:1) to afford the product as a brown oil (45.03 mg, 50%); ^1^H-NMR (500 MHz, CDCl_3_) δ 6.05 (s, 1H), 2.44 (t, *J* = 7.5 Hz, 4H), 1.69–1.61 (m, 4H), 0.95 (td, *J* = 7.4, 1.1 Hz, 6H) ppm; ^13^C-NMR (125 MHz, CDCl_3_) δ 180.6, 169.1, 113.0, 35.4, 20.1, 13.3 ppm; IR (KBr): 3437, 2965, 2875, 1663, 1619, 1411, 1398, 1148, 933, 864 cm^−1^; HRMS (*m*/*z*) (APCI): calcd. for C_11_H_14_O_2_ 181.1230 [M + H^+^]; found 181.1221.

*2,6-Dicyclopropyl-4H-pyran-4-one* (**2j**) [78]. The general procedure was used with 1,5-dicyclopropylpenta-1,4-diyn-3-one (79.04 mg, 0.5 mmol, 1 equiv.) and water (1 mL). The crude obtained was purified by column chromatography (petroleum ether/ethyl acetate = 5:1 to 2:1) to afford the product as a white solid (50.19 mg, 57%); m.p. 146.7–150.7 °C; ^1^H-NMR (500 MHz, CDCl_3_) δ 6.04 (s, 2H), 1.72 (tt, *J* = 8.3, 5.0 Hz, 2H), 1.00–0.95 (m, 4H), 0.92–0.88 (m, 4H) ppm; ^13^C-NMR (125 MHz, CDCl_3_) δ 179.5, 168.6, 111.1, 13.7, 7.8 ppm; IR (KBr): 3045, 3010, 2955, 1655, 1602, 1586, 1401, 1095, 1053, 858 cm^−1^; HRMS (*m*/*z*) (APCI): calcd. for C_11_H_13_O_2_ 177.0917 [M + H^+^]; found 177.0908.

*2-Phenyl-6-propyl-4H-pyran-4-one* (**2k**) [78]. The general procedure was used with 1-phenylocta-1,4-diyn-3-one (98.04 mg, 0.5 mmol, 1 equiv.) and water (1 mL). The crude obtained was purified by column chromatography (petroleum ether/ethyl acetate = 5:1 to 2:1) to afford the product as a brown solid (83.50 mg, 78%); m.p. 49.8–51.5 °C; ^1^H-NMR (500 MHz, CDCl_3_) δ 7.75 (dd, *J* = 7.7, 1.8 Hz, 1H), 7.51–7.46 (m, 1H), 6.72 (s, 1H), 6.19 (s, 1H), 2.60 (t, *J* = 7.5 Hz, 1H), 1.82–1.73 (m, 1H), 1.03 (t, *J* = 7.4 Hz, 1H) ppm; ^13^C-NMR (125 MHz, CDCl_3_) δ 180.1, 168.8, 163.6, 131.5, 131.3, 129.0, 125.8, 114.0, 111.1, 35.6, 20.3, 13.5 ppm; IR (KBr): 3060, 2926, 1653, 1617, 1493, 1450, 1409, 1061, 937, 866, 772, 691 cm^−1^; HRMS (*m*/*z*) (APCI): calcd. for C_14_H_15_O_2_ 215.1074 [M + H^+^]; found 215.1065.

*2-Phenyl-6-(p-tolyl)-4H-pyran-4-one* (**2l**) [83]. The general procedure was used with 1-phenyl-5-(*p*-tolyl)penta-1,4-diyn-3-one (112.04 mg, 0.5 mmol, 1 equiv.) and water (1 mL). The crude obtained was purified by column chromatography (petroleum ether/ethyl acetate = 5:1 to 2:1) to afford the product as a yellow solid (85.18 mg, 65%); m.p. 155.1–156.4 °C (lit: 150 °C); ^1^H-NMR (500 MHz, CDCl_3_) δ 7.88–7.83 (m, 2H), 7.75 (d, *J* = 8.2 Hz, 2H), 7.54–7.51 (m, 3H), 7.32 (d, *J* = 8.1 Hz, 2H), 6.83–6.78 (m, 2H), 2.44 (s, 3H) ppm; ^13^C-NMR (125 MHz, CDCl_3_) δ 180.4, 163.6, 163.3, 142.0, 131.5, 131.4, 129.8, 129.1, 128.6, 125.91, 125.86, 111.3, 110.7, 21.5 ppm; IR (KBr): 3064, 2922, 2854, 1646, 1606, 1448, 1413, 1387, 943, 816 cm^−1^; HRMS (*m*/*z*) (APCI): calcd. for C_18_H_15_O_2_ 263.1074 [M + H^+^]; found 263.1061.

*2-(4-Methoxyphenyl)-6-phenyl-4H-pyran-4-one* (**2m**) [78]. The general procedure was used with 1-(4-methoxyphenyl)-5-phenylpenta-1,4-diyn-3-one (130.04 mg, 0.5 mmol, 1 equiv.) and water (1 mL). The crude obtained was purified by column chromatography (petroleum ether/ethyl acetate = 5:1 to 2:1) to afford the product as a brown solid (97.33 mg, 70%); m.p. 161.3–162.2 °C (lit: 162 °C); ^1^H-NMR (500 MHz, CDCl_3_) δ 7.82 (dd, *J* = 6.6, 3.0 Hz, 2H), 7.78 (d, *J* = 8.9 Hz, 2H), 7.50 (dd, *J* = 5.0, 1.7 Hz, 3H), 7.00 (d, *J* = 8.9 Hz, 2H), 6.76 (d, *J* = 1.7 Hz, 1H), 6.70 (d, *J* = 1.7 Hz, 1H), 3.86 (s, 3H) ppm; ^13^C-NMR (125 MHz, CDCl_3_) δ 180.2, 163.3, 163.0, 162.2, 131.5, 131.2, 129.0, 127.5, 125.8, 123.6, 114.5, 111.1, 109.8, 55.4 ppm; IR (KBr): 3443, 3067, 2900, 2843, 1647, 1604, 1509, 1448, 1423, 1383, 1023, 832, 767, 684 cm^−1^; HRMS (*m*/*z*) (APCI): calcd. for C_18_H_15_O_3_ 279.1014 [M + H^+^]; found 279.1013.

*2-(4-Fluorophenyl)-6-phenyl-4H-pyran-4-one* (**2n**). The general procedure was used with 1-(4-fluorophenyl)-5-phenylpenta-1,4-diyn-3-one (124.03 mg, 0.5 mmol, 1 equiv.) and water (1 mL). The crude obtained was purified by column chromatography (petroleum ether/ethyl acetate = 5:1 to 2:1) to afford the product as a yellow solid (77.16 mg, 58%); m.p. 145.5–150.6 °C; ^1^H-NMR (500 MHz, CDCl_3_) δ 7.89–7.82 (m, 4H), 7.56–7.51 (m, 3H), 7.22 (t, *J* = 8.5 Hz, 2H), 6.82 (d, *J* = 1.8 Hz, 1H), 6.77 (d, *J* = 1.8 Hz, 1H) ppm; ^13^C-NMR (125 MHz, CDCl_3_) δ 180.1, 165.6, 163.6, 163.4, 162.5, 131.5, 131.3, 129.2, 128.1 (d, *J* = 8.9 Hz), 127.6, 125.9, 116.4 (d, *J* = 22.1 Hz), 111.3 (d, *J* = 24.2 Hz) ppm; IR (KBr): 3061, 2924, 1659, 1505, 1508, 1417, 1449, 1388, 1232, 1162 cm^−1^; HRMS (*m*/*z*) (APCI): calcd. for C_17_H_12_FO_2_ 267.0823 [M + H^+^]; found 267.0813.

*2-Cyclopropyl-6-phenyl-4H-pyran-4-one* (**2o**) [84]. The general procedure was used with 1-cyclopropyl-5-phenylpenta-1,4-diyn-3-one (97.04 mg, 0.5 mmol, 1 equiv.) and water (1 mL). The crude obtained was purified by column chromatography (petroleum ether/ethyl acetate = 5:1 to 2:1) to afford the product as a yellow solid (58.33 mg, 55%); m.p. 106.5–107.8 °C (lit: 106 °C); ^1^H-NMR (500 MHz, CDCl_3_) δ 7.67 (dd, *J* = 7.9, 1.7 Hz, 2H), 7.50–7.45 (m, 3H), 6.69 (d, *J* = 2.1 Hz, 1H), 6.23 (d, *J* = 2.1 Hz, 1H), 1.90 (tt, *J* = 7.9, 5.4 Hz, 1H), 1.12 (tt, *J* = 4.7, 2.5 Hz, 4H) ppm; ^13^C-NMR (125 MHz, CDCl_3_) δ 179.8, 169.5, 162.7, 131.3, 131.2, 129.0, 125.6, 111.6, 111.0, 14.1, 8.5 ppm; IR (KBr): 3059, 2927, 1651, 1609, 1544, 1496, 1448, 1394, 1253, 1193, 1087, 931, 878, 766, 685 cm^−1^; HRMS (*m*/*z*) (APCI): calcd. for C_14_H_13_O_2_ 213.0917 [M + H^+^]; found 213.0908.

*2-(2-Chlorophenyl)-6-phenyl-4H-pyran-4-one* (**2p**) [85]. The general procedure was used with 1-(2-chlorophenyl)-5-phenylpenta-1,4-diyn-3-one (132.02 mg, 0.5 mmol, 1 equiv.) and water (1 mL). The crude obtained was purified by column chromatography (petroleum ether/ethyl acetate = 5:1 to 2:1) to afford the product as a yellow solid (86.03 mg, 61%); m.p. 123.5–124.6 °C (lit: 122–124 °C); ^1^H-NMR (400 MHz, CDCl_3_) δ 7.86–7.81 (m, 2H), 7.60 (dd, *J* = 7.5, 1.8 Hz, 1H), 7.57–7.54 (m, 1H), 7.52–7.48 (m, 2H), 7.48–7.46 (m, 1H), 7.44 (dd, *J* = 6.6, 1.7 Hz, 1H), 7.41 (dd, *J* = 7.4, 1.4 Hz, 1H), 6.86 (d, *J* = 2.2 Hz, 1H), 6.67 (d, *J* = 2.2 Hz, 1H) ppm; ^13^C-NMR (100 MHz, CDCl_3_) δ 178.0, 164.1, 162.6, 132.8, 131.9, 131.5, 131.4, 131.2, 130.9, 130.7, 129.1, 127.2, 126.0, 116.8, 111.2 ppm; IR (KBr): 3059, 2931, 1667, 1650, 1600, 1580, 1403, 1250, 1000, 910, 665 cm^−1^; HRMS (*m*/*z*) (ESI): calcd. for C_17_H_12_ClO_2_ 283.0528 [M + H^+^]; found 283.0513.

*2-(4-Fluorophenyl)-6-(4-methoxyphenyl)-4H-pyran-4-one* (**2q**) [85]. The general procedure was used with 1-(4-fluorophenyl)-5-(4-methoxyphenyl)penta-1,4-diyn-3-one (139.04 mg, 0.5 mmol, 1 equiv.) and water (1 mL). The crude obtained was purified by column chromatography (petroleum ether/ethyl acetate = 5:1 to 2:1) to afford the product as a yellow solid (93.27 mg, 63%); m.p. 138.7–140.5 °C (lit: 144–148 °C); ^1^H-NMR (400 MHz, CDCl_3_) δ 7.86–7.82 (m, 2H), 7.80–7.77 (m, 2H), 7.27–7.18 (m, 2H), 7.05–7.00 (m, 2H), 6.72 (dd, *J* = 3.7, 1.9 Hz, 2H), 3.89 (s, 3H) ppm; ^13^C-NMR (100 MHz, CDCl_3_) δ 180.2, 165.8, 163.4, 163.3, 162.2 (d, *J* = 8.9 Hz), 128.1 (d, *J* = 8.8 Hz), 127.8 (d, *J* = 3.3 Hz), 127.6, 123.6, 116.4 (d, *J* = 22.1 Hz), 114.6, 111.0, 109.9, 55.5 ppm; IR (KBr): 3673, 3067, 2969, 1657, 1610, 1509, 1422, 1385, 1270, 1227, 1169, 1074, 1021, 841 cm^−1^; HRMS (*m*/*z*) (ESI): calcd. for C_18_H_14_FO_3_ 297.0929 [M + H^+^]; found 297.0913.

*2-Phenyl-4H-pyran-4-one* (**2r**) [86]. The general procedure was used with 1-phenylpenta-1,4-diyn-3-one (77.02 mg, 0.5 mmol, 1 equiv.) and water (1 mL). The crude obtained was purified by column chromatography (petroleum ether/ethyl acetate = 5:1 to 2:1) to afford the product as a yellow solid (43.02 mg, 50%); yellow solid; m.p. 102.2–103.5 °C (lit: 100–102 °C); ^1^H-NMR (400 MHz, CDCl_3_) δ 7.84 (d, *J* = 5.8 Hz, 1H), 7.74 (dd, *J* = 7.9, 1.7 Hz, 2H), 7.51–7.44 (m, 3H), 6.78 (d, *J* = 2.3 Hz, 1H), 6.38 (dd, *J* = 5.8, 2.3 Hz, 1H) ppm; ^13^C-NMR (100 MHz, CDCl_3_) δ 179.0, 163.9, 154.8, 131.4, 131.0, 129.0, 125.7, 117.0, 112.3 ppm; IR (KBr): 3090, 1675, 1650, 1590, 1549, 1490, 1450, 1402, 1350, 1050, 931, 875, 795, 730, 650 cm^−1^; HRMS (*m*/*z*) (ESI): calcd. for C_11_H_9_O_2_ 173.0604 [M + H^+^]; found 173.0603.

### 3.3. Control Experiments

#### 3.3.1. Deuterium Labeling Experiments

**Scheme 6:**
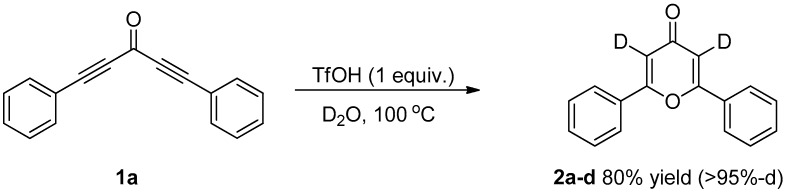
Deuterium Labeling Experiments.

The reaction mixture of **1** (0.5 mmol), TfOH (1 equiv.), and D_2_O (1 mL) in a 15 mL test tube was stirred at 100 °C for 36 h, and monitored periodically by TLC. Upon completion, the reaction mixture was diluted with water (5 mL) and extracted with ethyl acetate (3 × 5 mL). The combined organic layers were washed with water and brine, dried over MgSO_4_ and filtered. The solvent was removed under vacuum. The residue was purified by flash column chromatography (petroleum ether and ethyl acetate, *v*/*v* = 5:1 to 2:1) to afford 4-pyrone **2a-d** (100.04 mg, 80%) as a yellow solid; m.p. 116.1–119.5 °C; ^1^H-NMR (500 MHz, CDCl_3_) δ 7.90–7.83 (m, 4H), 7.55–7.51 (m, 6H), 6.84 (s, 0.12H) (Scheme 6).

#### 3.3.2. O^18^-Labelling Experiment

**Scheme 7:**
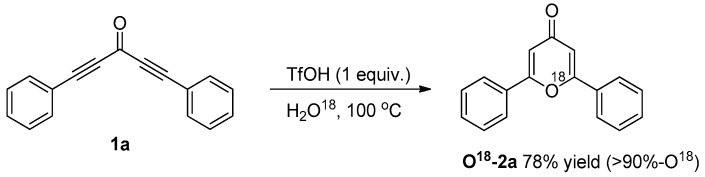
O^18^-Labelling Experiment.

The reaction mixture of **1a** (0.5 mmol), TfOH (1 equiv.), and H_2_O^18^ (1 mL) in a 15 mL test tube was stirred at 100 °C for 36 h, and monitored periodically by TLC. Upon completion, the reaction mixture was diluted with water (5 mL) and extracted with ethyl acetate (3 × 5 mL). The combined organic layers were washed with water and brine, dried over MgSO_4_ and filtered. The solvent was removed under vacuum. The residue was purified by flash column chromatography (petroleum ether and ethyl acetate, *v*/*v* = 5:1 to 2:1) to afford 4-pyrone **O^18^-2a** (78%) (Scheme 7).

#### 3.3.3. Gram-Scale Synthesis

**Scheme 8:**
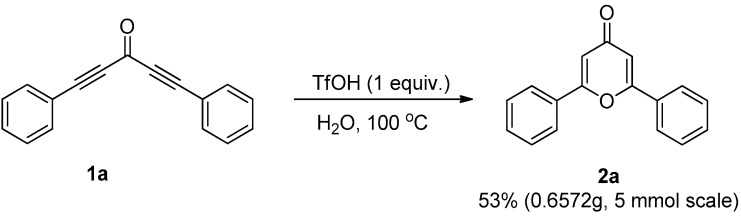
Gram-Scale Synthesis.

The reaction mixture of **1a** (5 mmol), TfOH (1 equiv.) and H_2_O (10 mL) in a 50 mL round-bottom flask was stirred at 100 °C for 36 h, and monitored periodically by TLC. Upon completion, the reaction mixture was diluted with water (30 mL) and extracted with ethyl acetate (3 × 30 mL). The combined organic layers were washed with water and brine, dried over MgSO_4_ and filtered. The solvent was removed under vacuum. The residue was purified by flash column chromatography (petroleum ether and ethyl acetate, *v*/*v* = 5:1 to 2:1) to afford 4-pyrone **2a** (53%) (Scheme 8).

## 4. Conclusions

We have developed a simple and efficient transition-metal-free method for the synthesis of substituted 4-pyrones from diynones and H_2_O. Water is a cheap, green and readily available staring material, which converted to the desired 4-pyrone products via a nucleophilic addition/cyclization/ dehydrogenation process. The operational simplicity, good yields, and environmentally benign nature of this method make it an attractive route to 4-pyrones. Further studies on the applications of 4-pyrones in drug design are currently ongoing in our laboratory.

## Figures and Tables

**Table 1:**
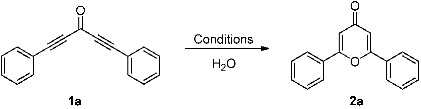
Optimization of reaction conditions ^a^.

Entry	Catalyst	Time (h)	Yield (%) ^b^
1	TfOH	24	70
2	CH_3_COOH	24	0
3	PTSA	24	50
4	HCl	24	0
5	H_3_PO_4_	24	0
6	PhCOOH	24	10
7 ^c^	TfOH	24	10
8 ^d^	TfOH	24	50
9 ^e^	TfOH	24	80
10 ^f^	TfOH	24	20
11 ^g^	TfOH	24	75
12	TfOH	36	83

^a^ Reaction conditions: **1a** (0.5 mmol), catalyst (1 equiv.), H_2_O (1 mL), at 100 °C; ^b^ Isolated yields; ^c^ TfOH (0.2 equiv.); ^d^ TfOH (0.5 equiv.); ^e^ TfOH (2 equiv.); ^f^ At 80 °C; ^g^ The reaction was carried out in a sealed tube at 130 °C.

## Supplementary Materials

Supplementary materials can be accessed at: http://www.mdpi.com/1420-3049/22/1/109/s1: copies of NMR spectra and HRMS spectra of products.

## Abbreviations

TfOH	trifluoromethanesulfonic acid
PTSA	4-methylbenzenesulfonic acid
HCl	hydrochloric acid
HIV	human immunodeficiency virus
HOAc	acetic acid
Ph	phenyl
Me	methyl
OMe	methoxyl
Et	ethyl
*^t^*Bu	tertiary butyl
*^n^*Pr	*n*-propyl
NMR	nuclear magnetic resonance
HRMS	high-resolution mass
