# Supplementary material for: Atom-Economic Synthesis of 4-Pyrones from Diynones and Water
Source: Molecules. 2017 Jan 10;22(1):109. doi: 10.3390/molecules22010109 (PMC6155647; doi:10.3390/molecules22010109)
Supplement: Supplementary file 1 [file molecules-22-00109-s001.pdf]

Supplementary Materials: Atom-Economic Synthesis of 4-Pyrones from Diynones and Water

Yan-Li Xu, Qing-Hu Teng, Wei Tong, Heng-Shan Wang, Ying-Ming Pan and Xian-Li Ma

1. Copies of ^{1}H-NMR and ^{13}C-NMR Spectra of Products

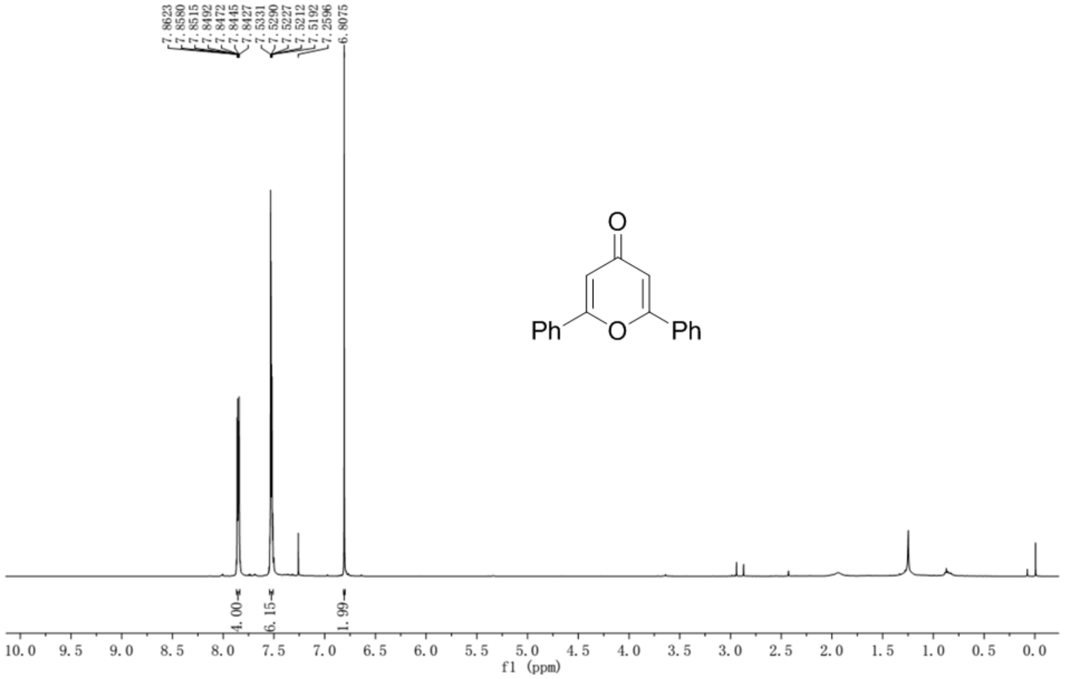

Figure S1. ^{1}H-NMR Spectra of **2a**.

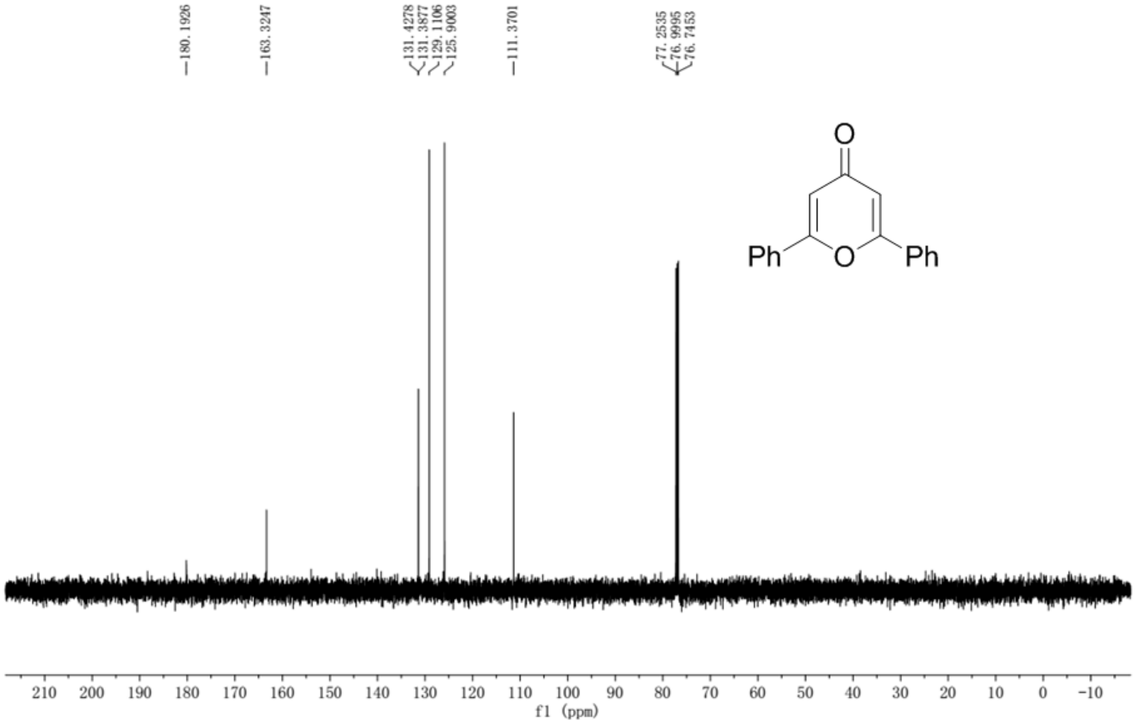

Figure S2. ^{13}C-NMR Spectra of **2a**.

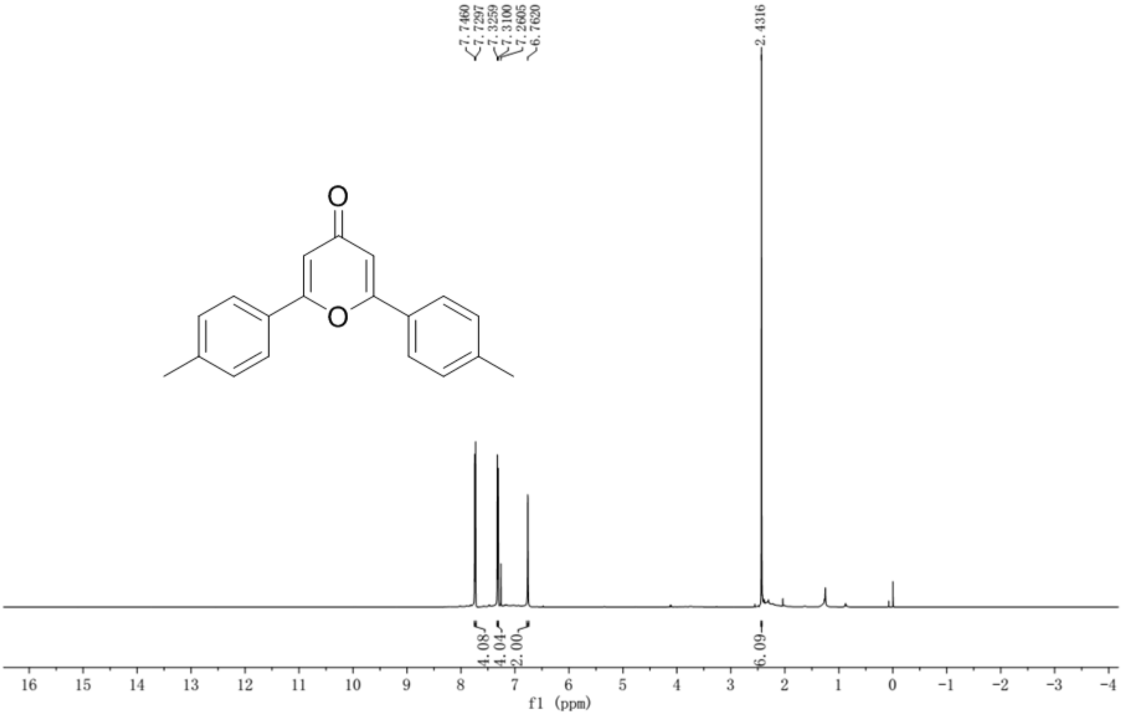

Figure S3. ^{1}H-NMR Spectra of **2b**.

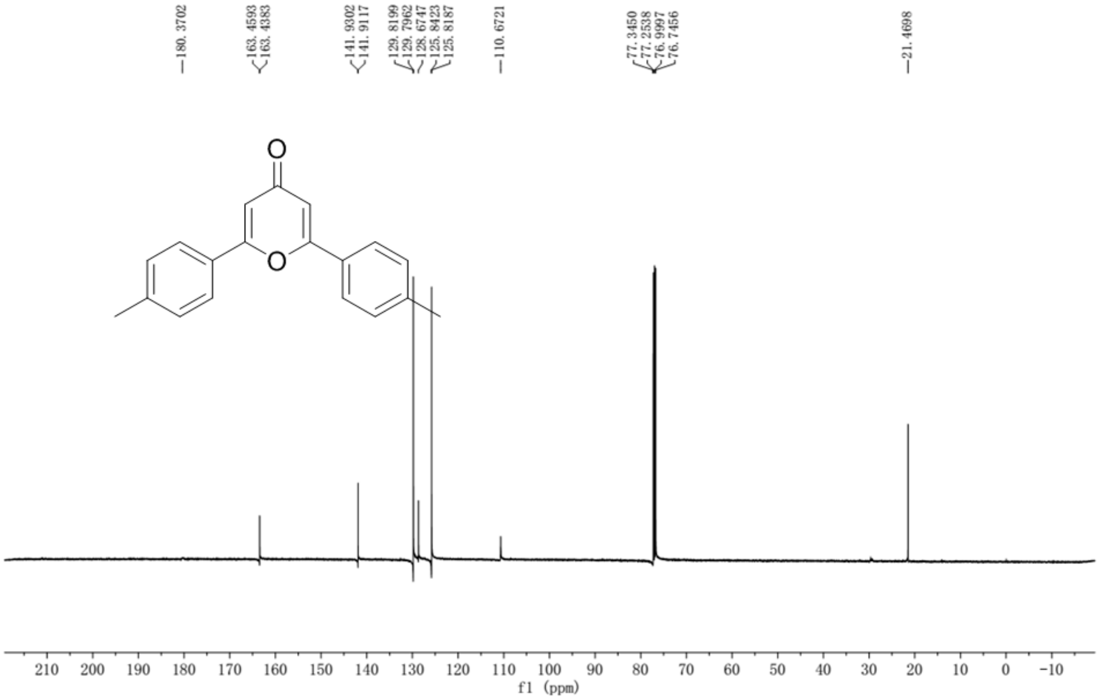

Figure S4. ^{13}C-NMR Spectra of **2b**.

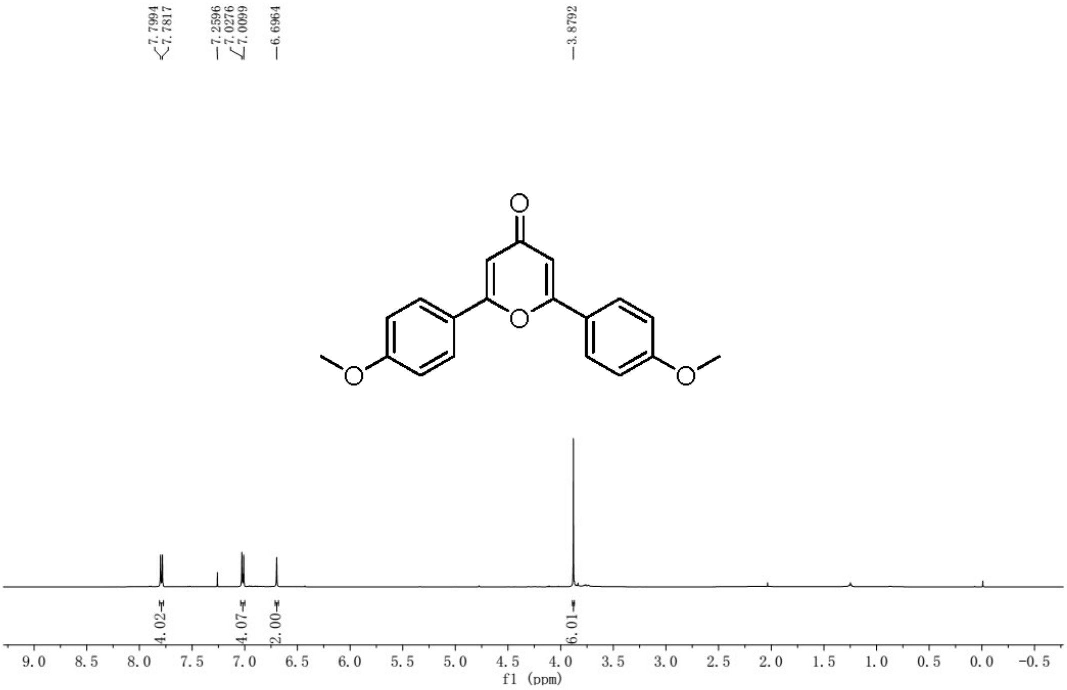

Figure S5. ^{1}H-NMR Spectra of **2c**.

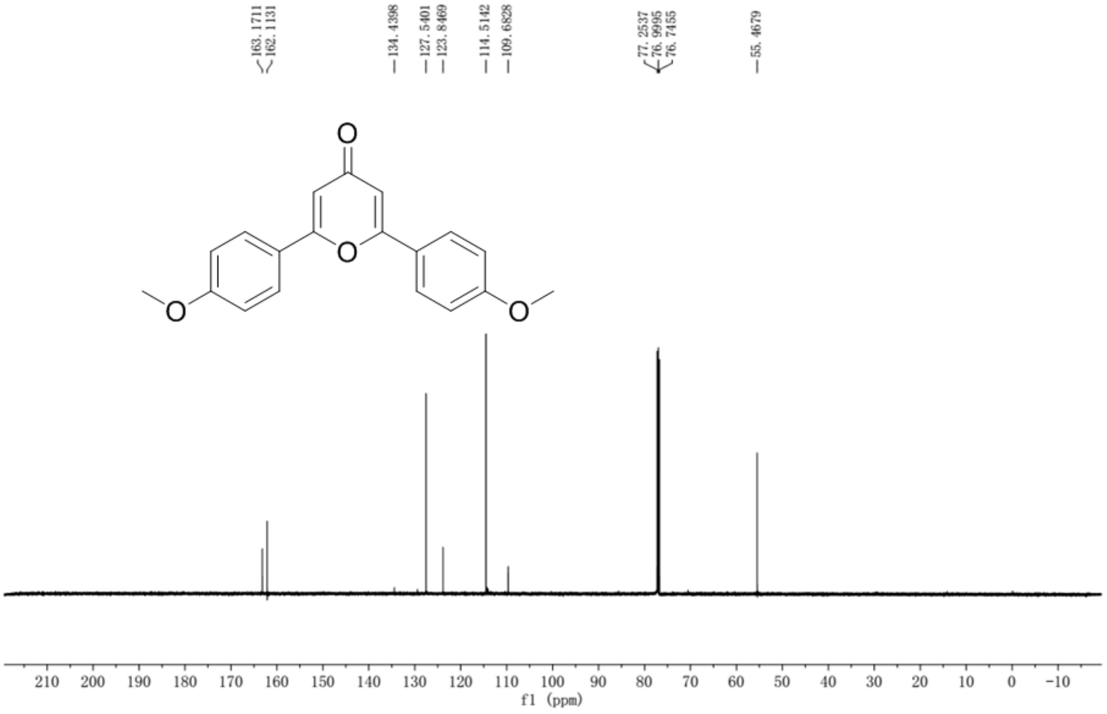

Figure S6. ^{13}C-NMR Spectra of **2c**.

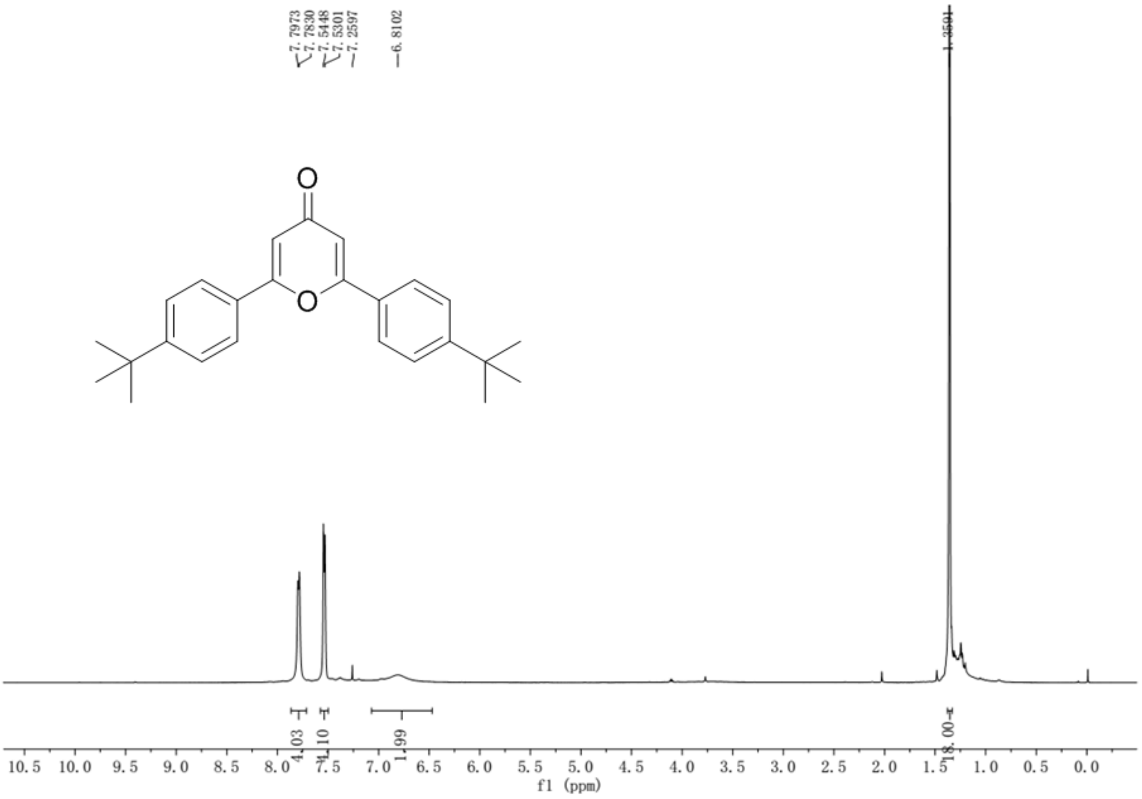

Figure S7. ^{1}H-NMR Spectra of **2d**.

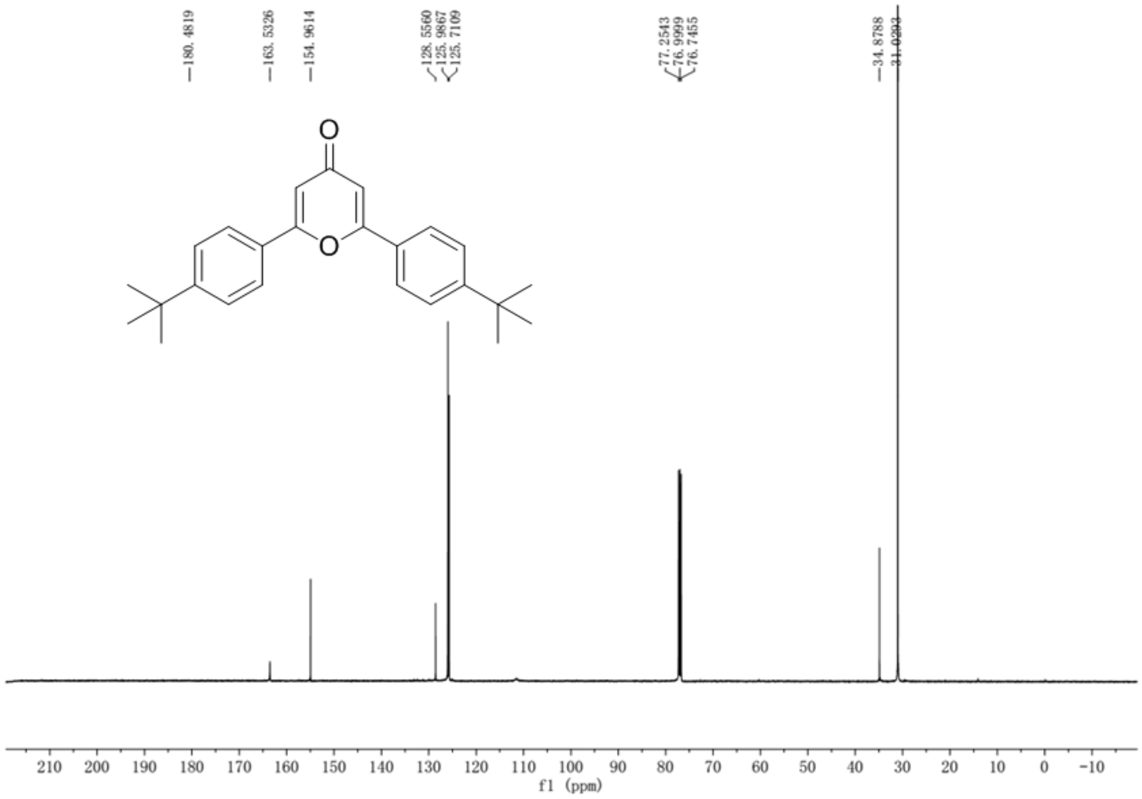

Figure S8. ^{13}C-NMR Spectra of **2d**.

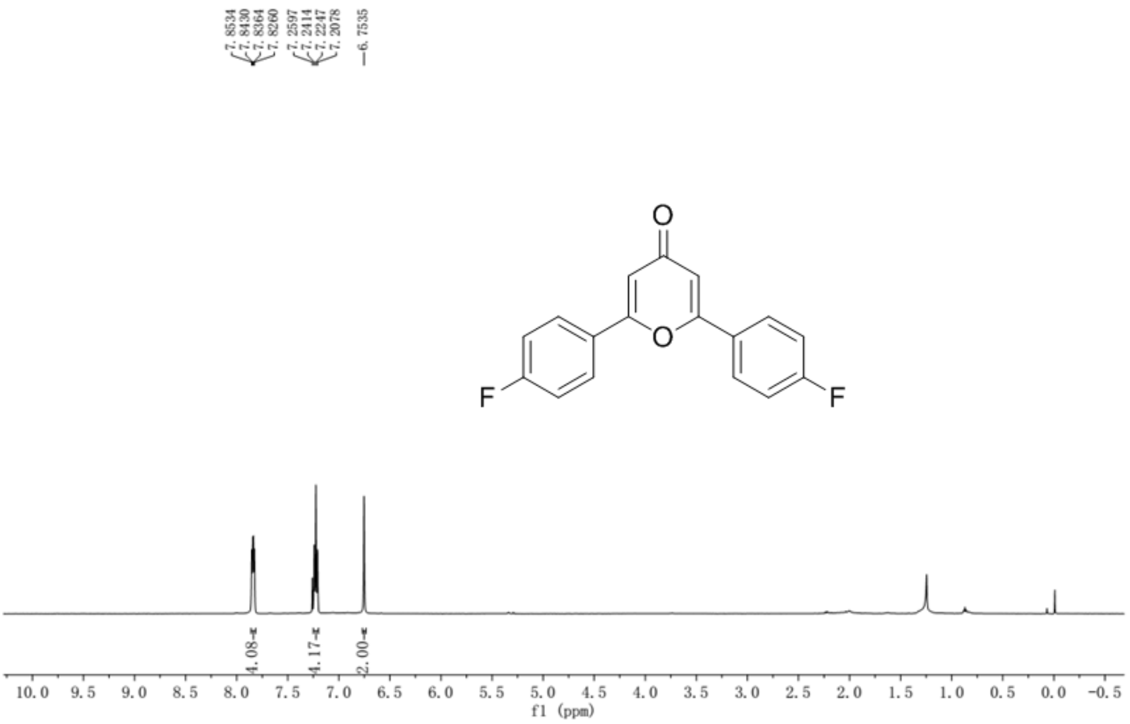

Figure S9. ^{1}H-NMR Spectra of **2e**.

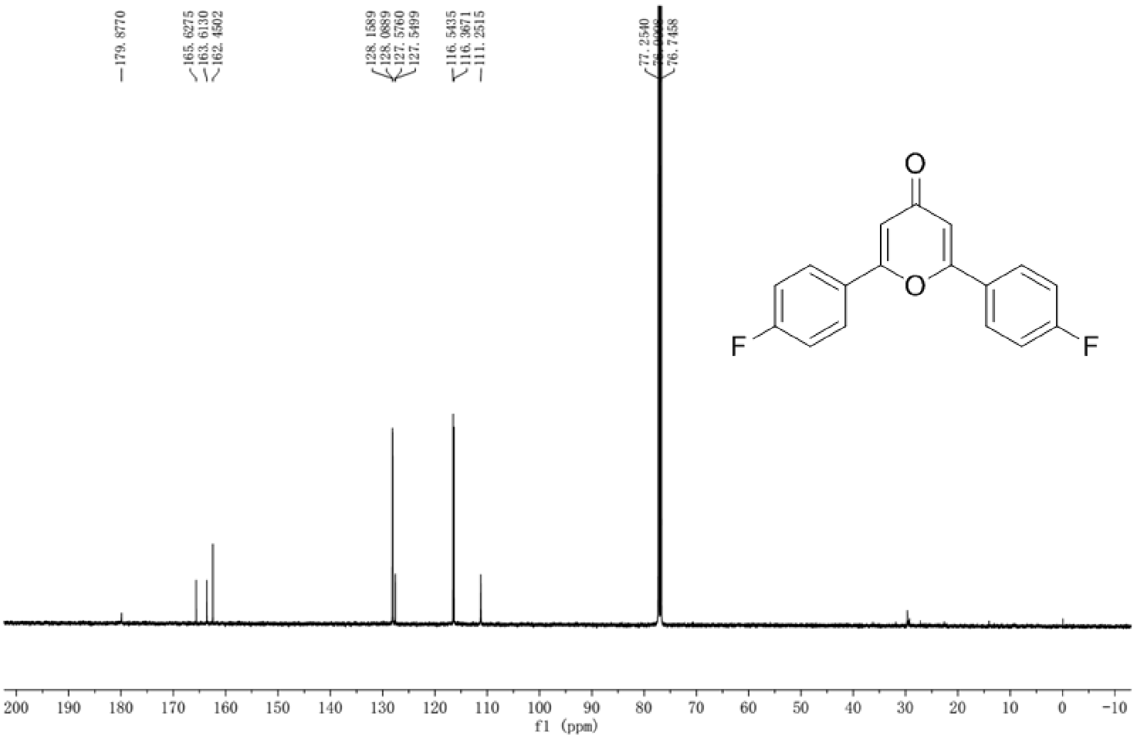

Figure S10. ^{13}C-NMR Spectra of **2e**.

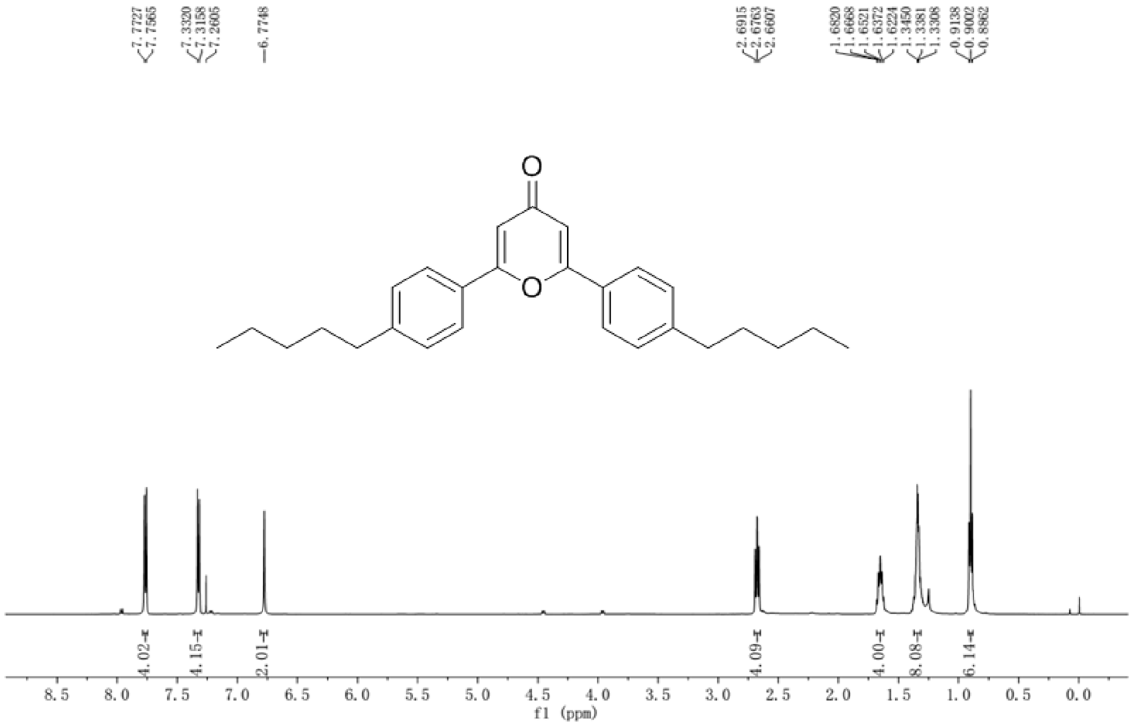

Figure S11. ^{1}H-NMR Spectra of **2f**.

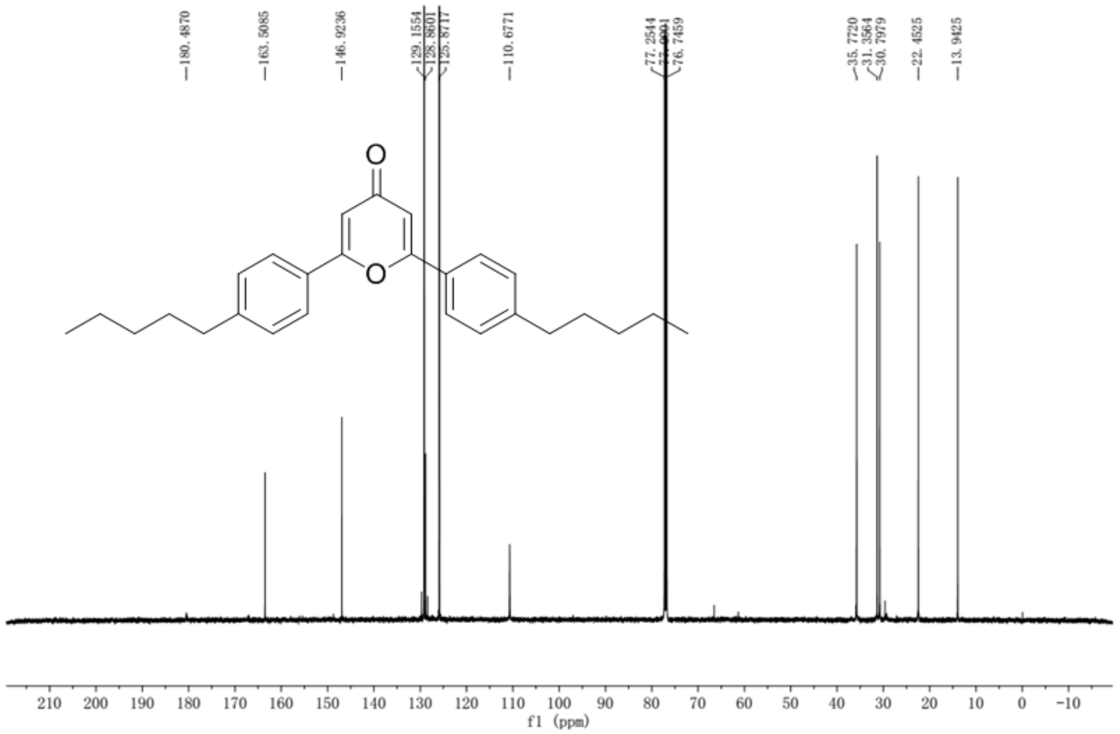

Figure S12. ^{13}C-NMR Spectra of **2f**.

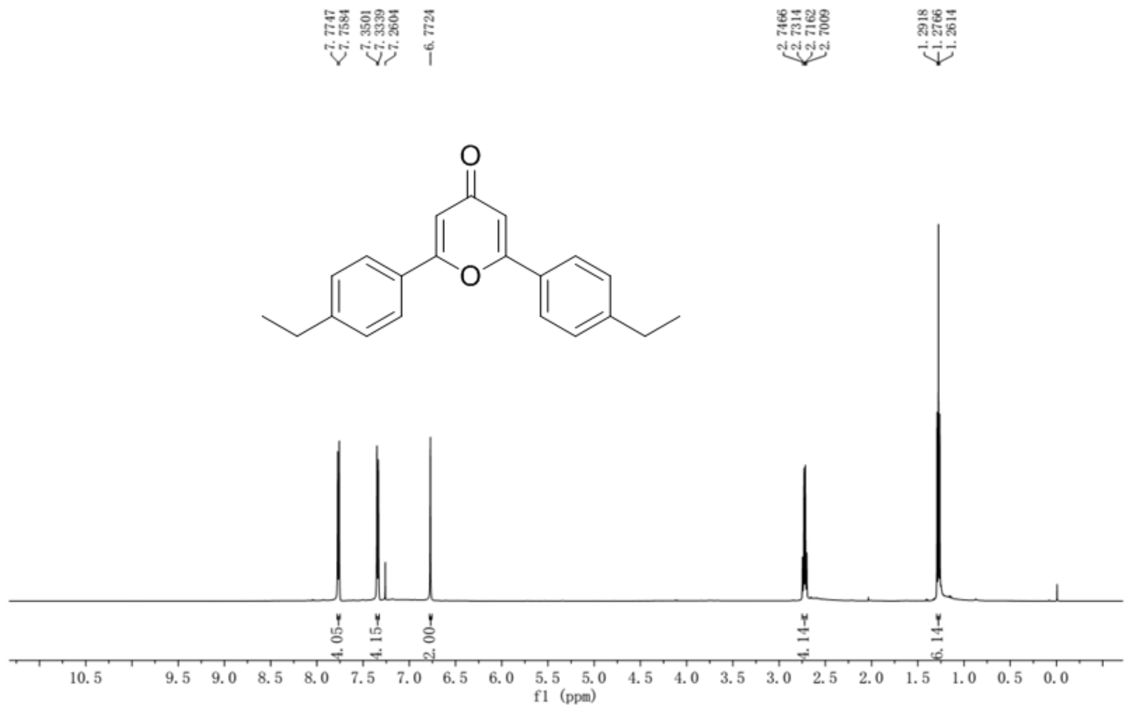

Figure S13. ^{1}H-NMR Spectra of **2g**.

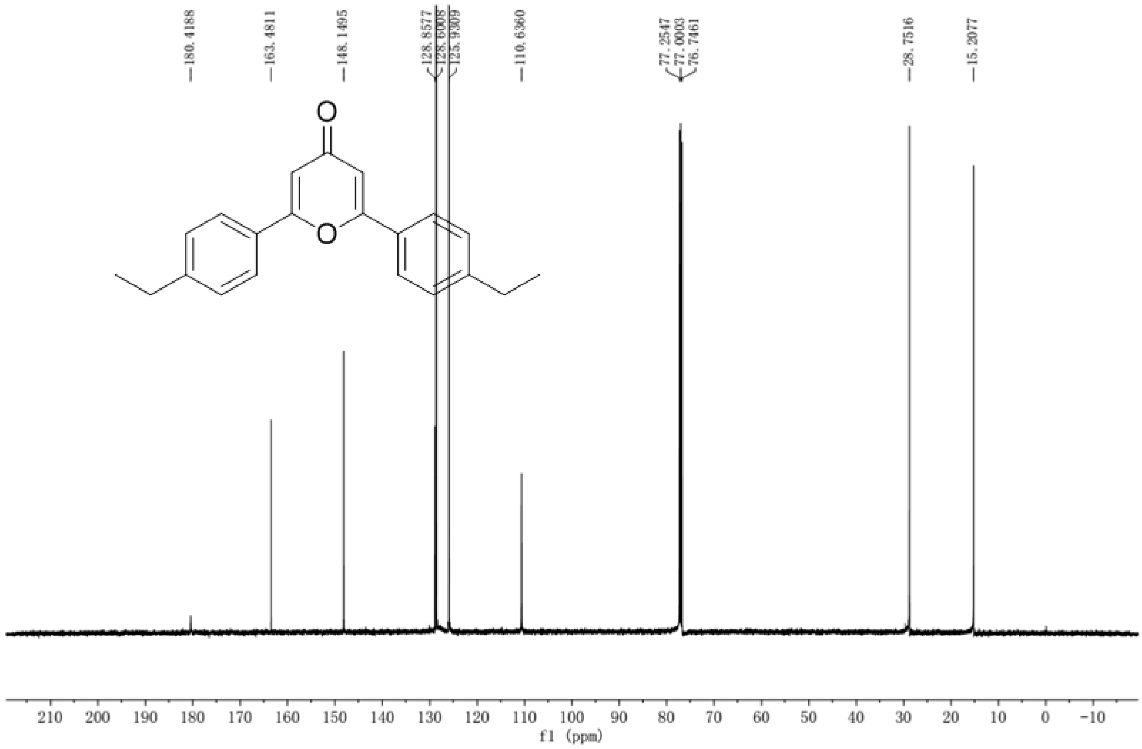

Figure S14. ^{13}C-NMR Spectra of **2g**.

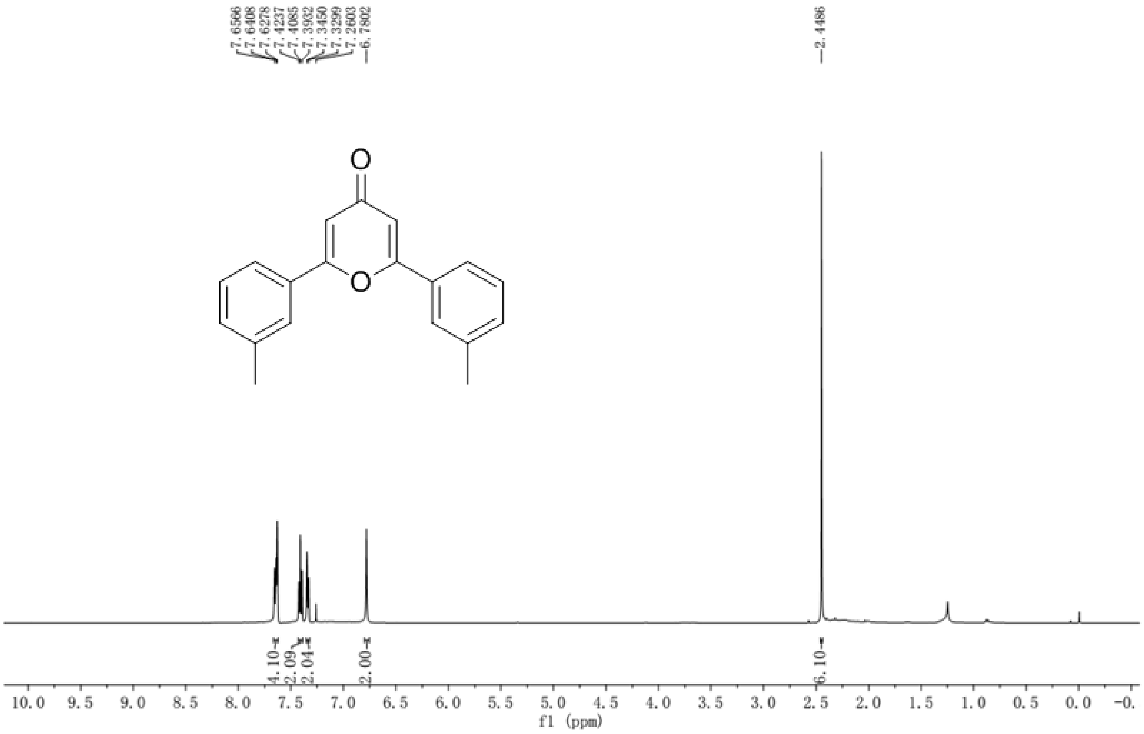

Figure S15. ¹H-NMR Spectra of **2h**.

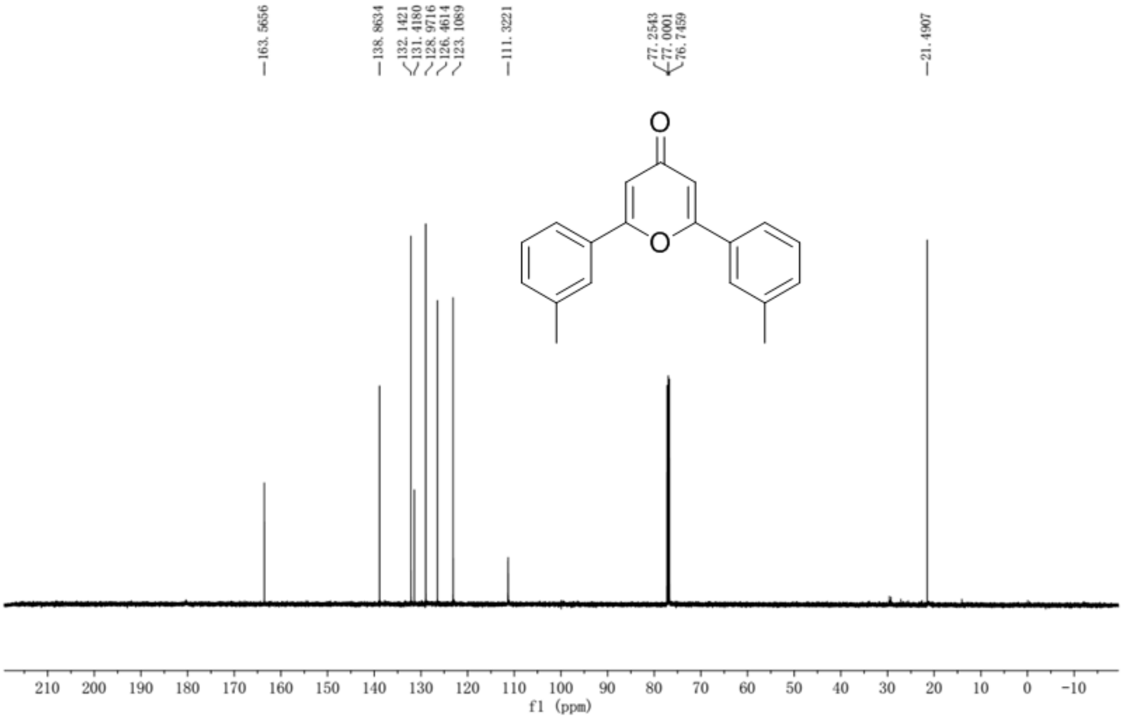

Figure S16. ¹³C-NMR Spectra of **2h**.

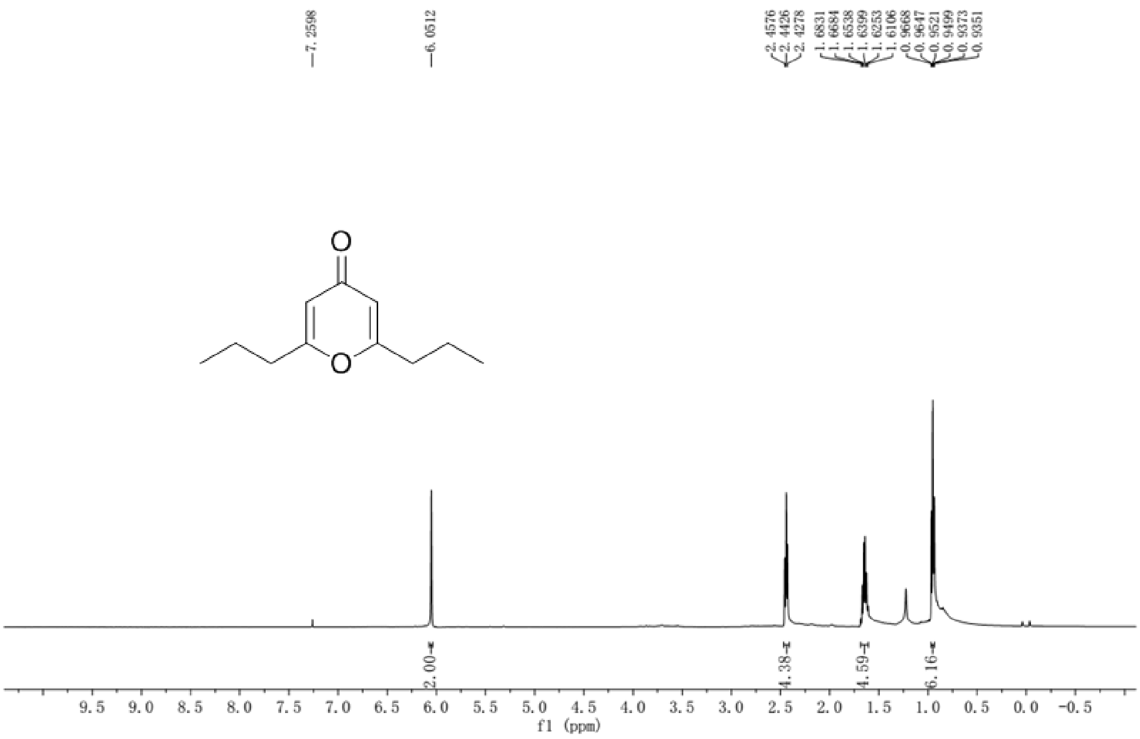

Figure S17. ^{1}H-NMR Spectra of **2i**.

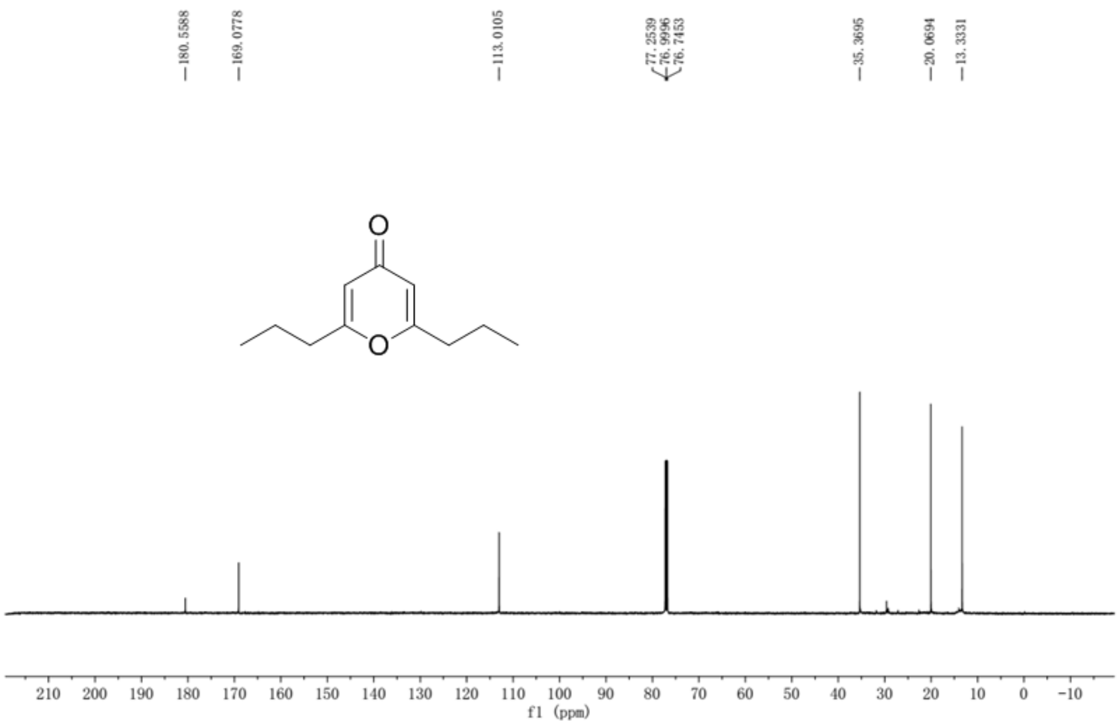

Figure S18. ^{13}C-NMR Spectra of **2i**.

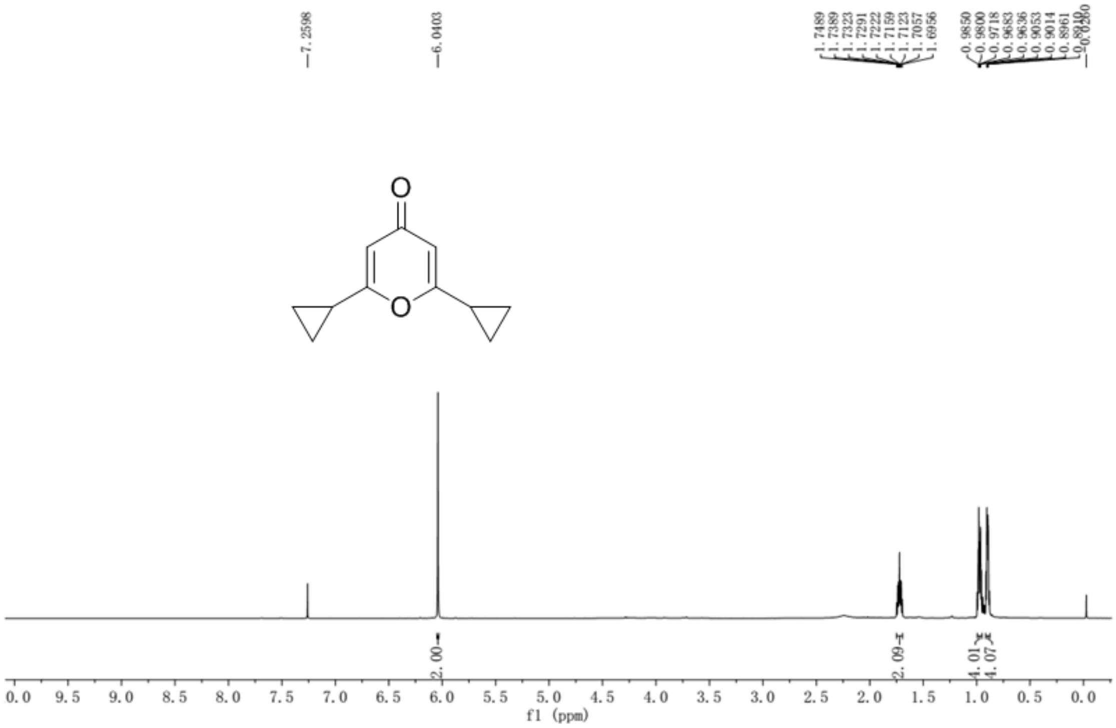

Figure S19. ^{1}H-NMR Spectra of **2j**.

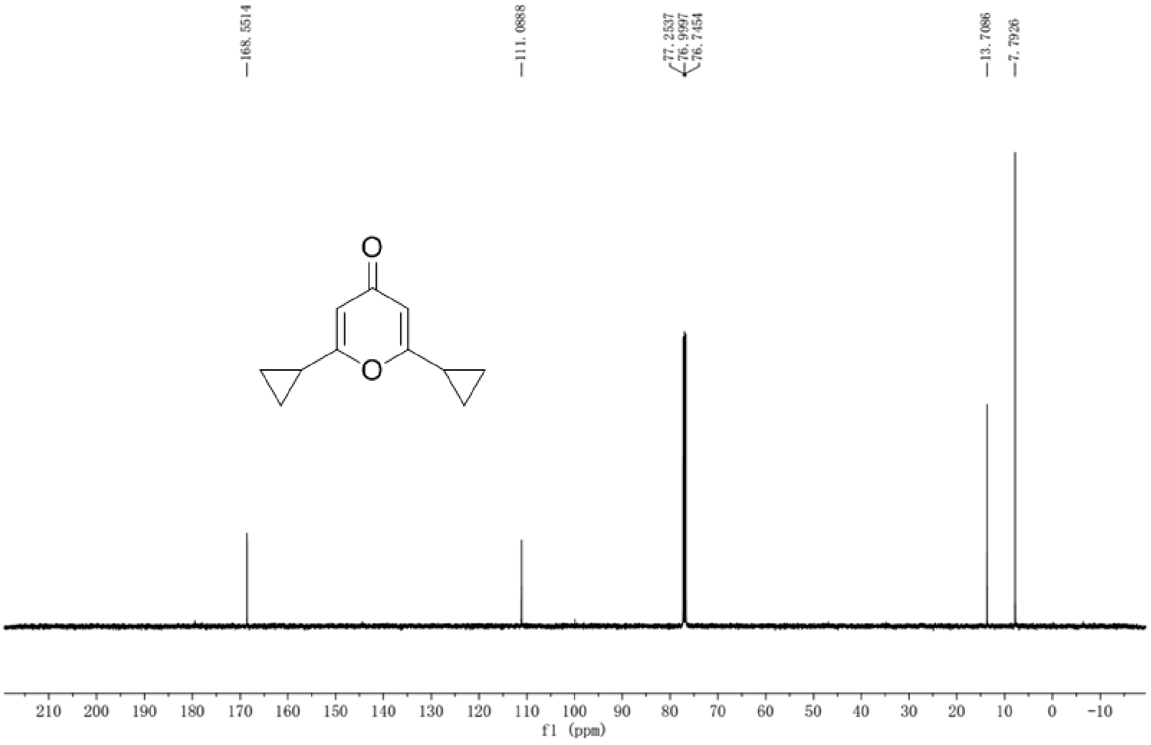

Figure S20. ^{13}C-NMR Spectra of **2j**.

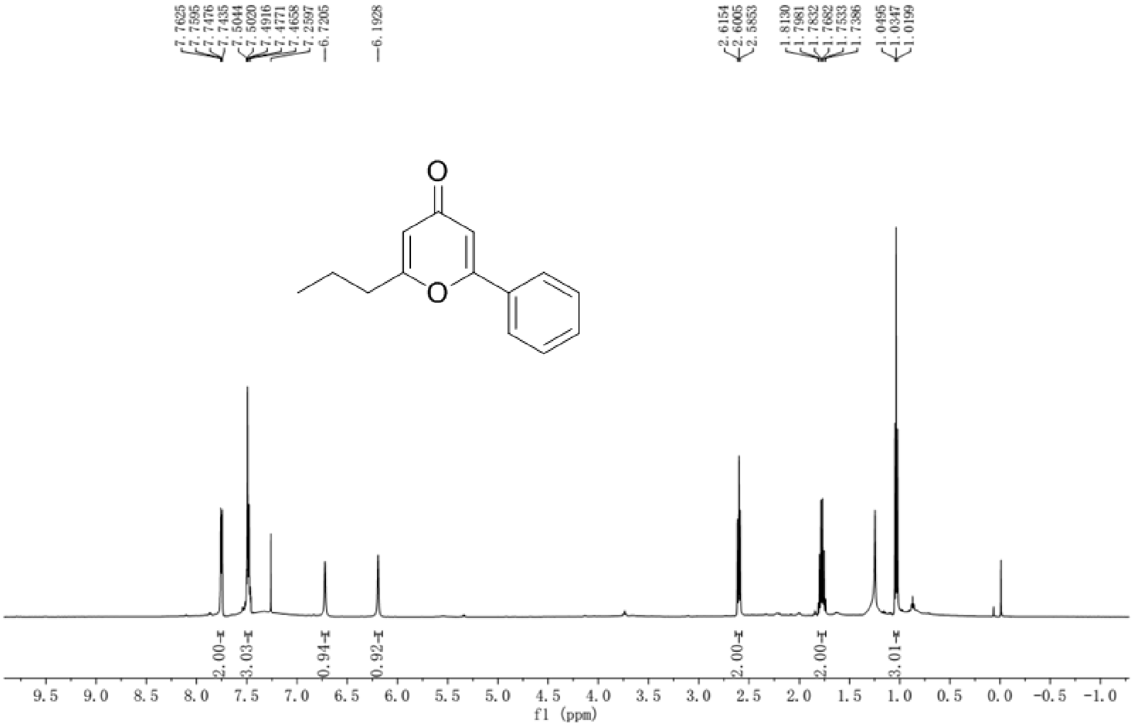

Figure S21. ^{1}H-NMR Spectra of **2k**.

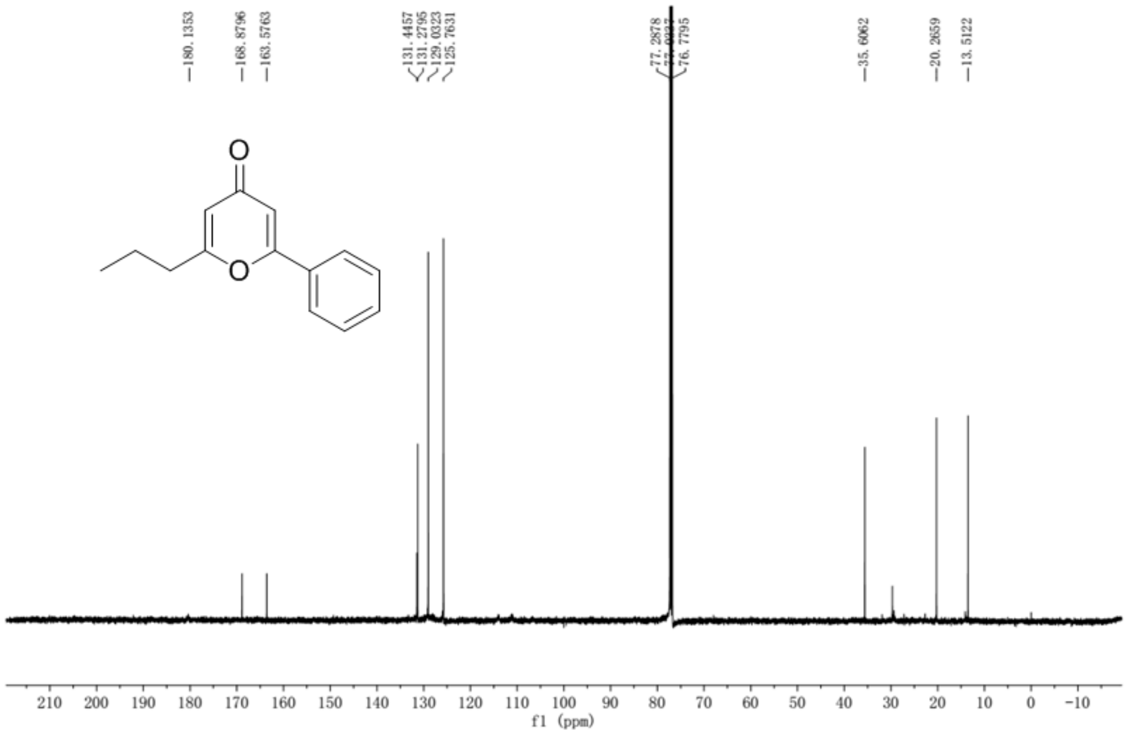

Figure S22. ^{13}C-NMR Spectra of **2k**.

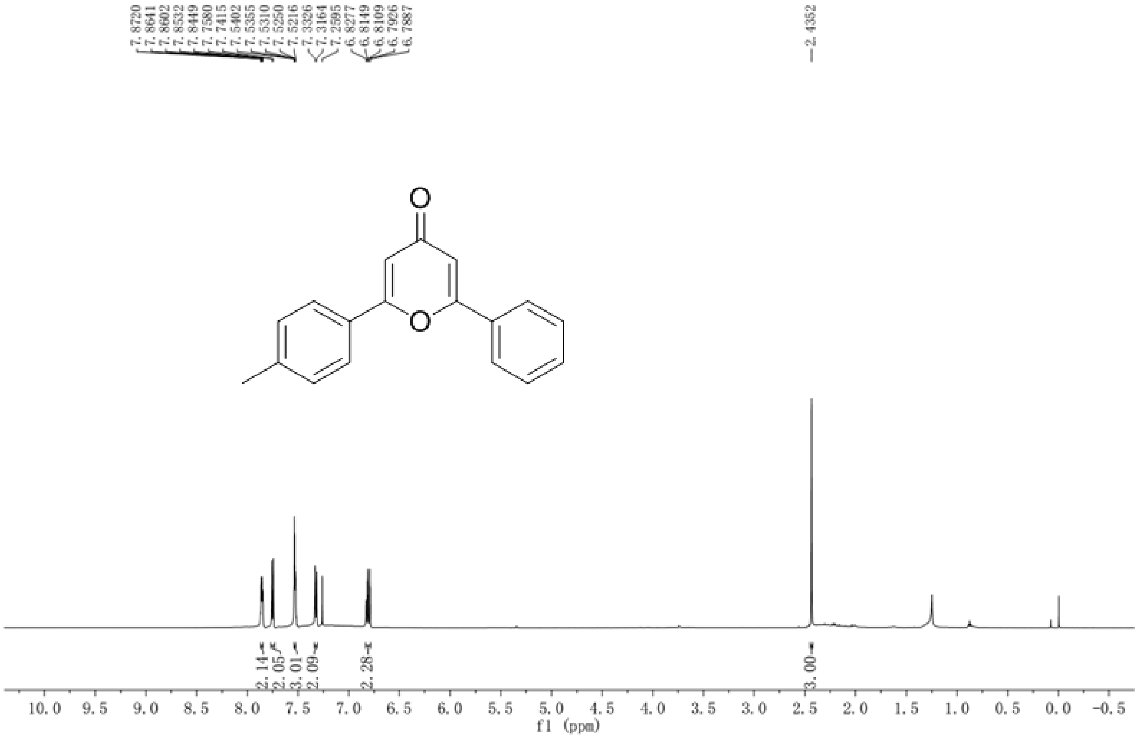

Figure S23. ^{1}H-NMR Spectra of **2l**.

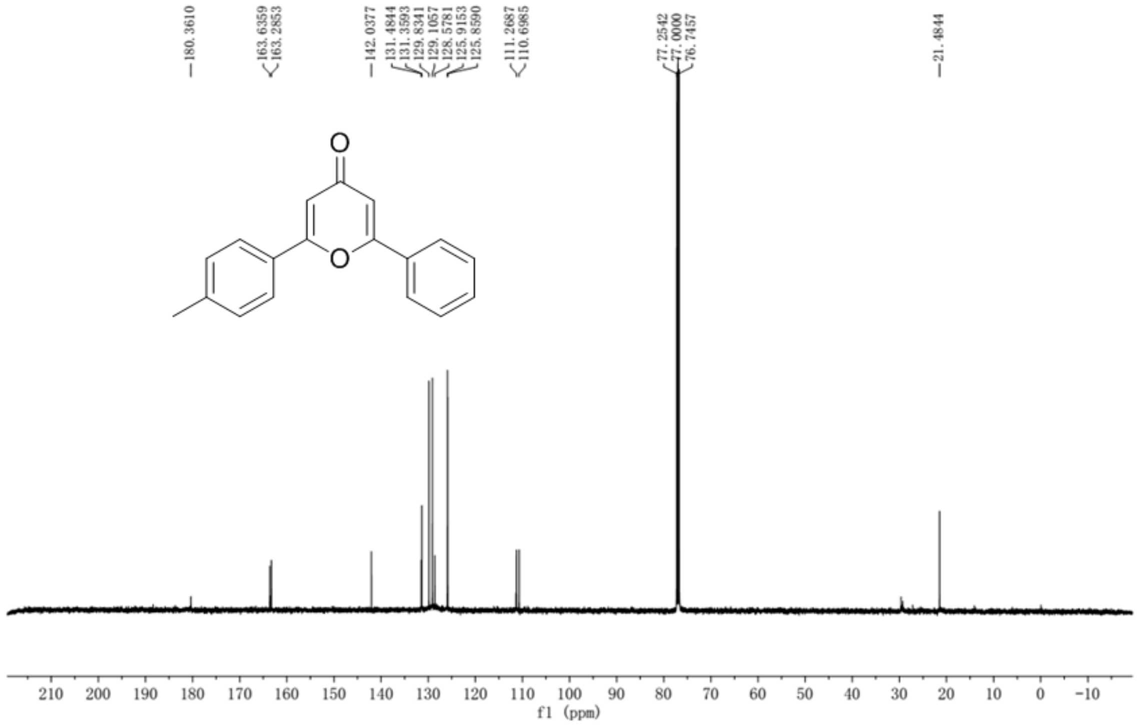

Figure S24. ^{13}C-NMR Spectra of **2l**.

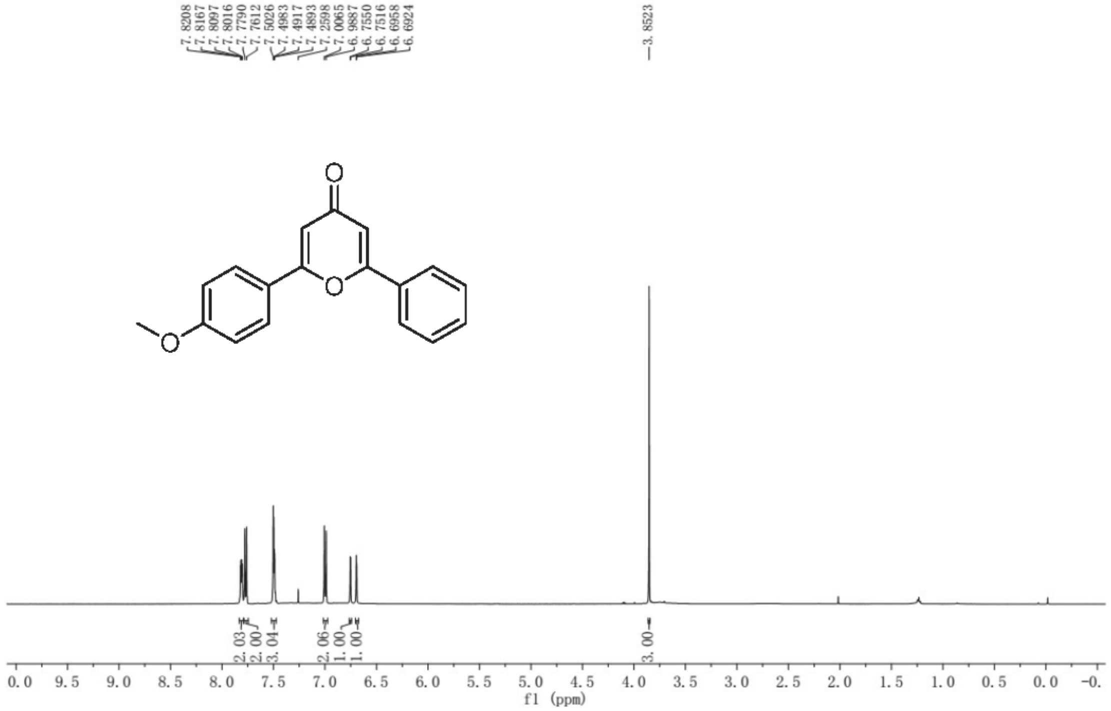

Figure S25. ^{1}H-NMR Spectra of **2m**.

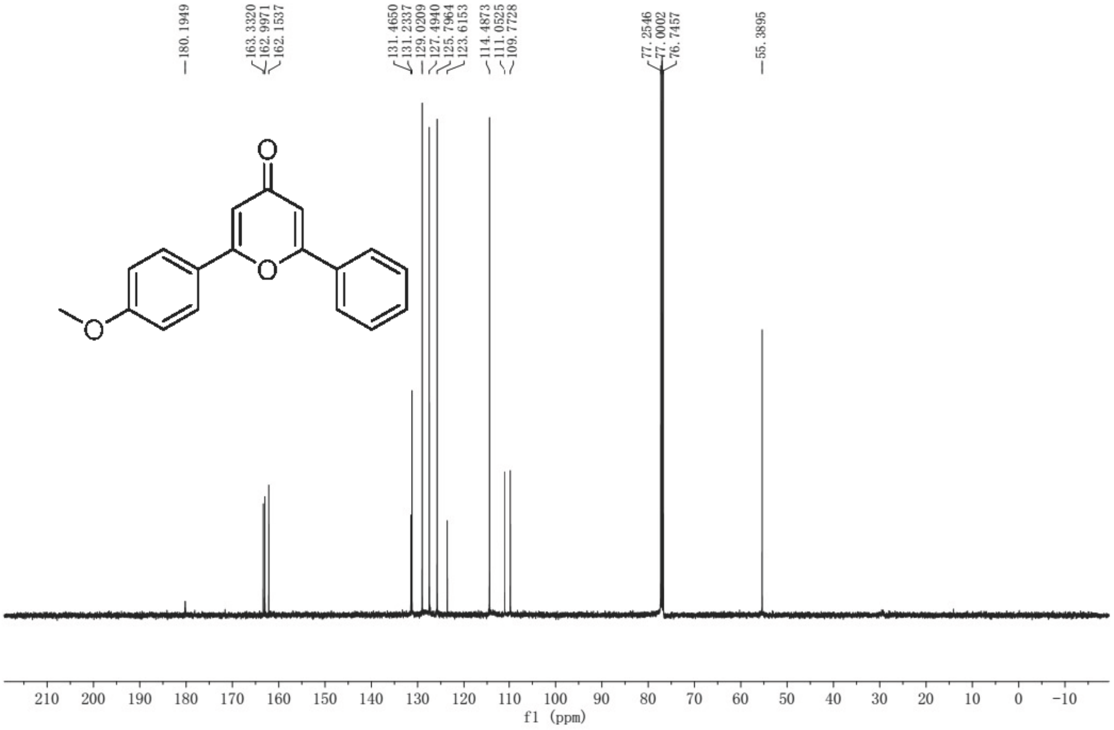

Figure S26. ^{13}C-NMR Spectra of **2m**.

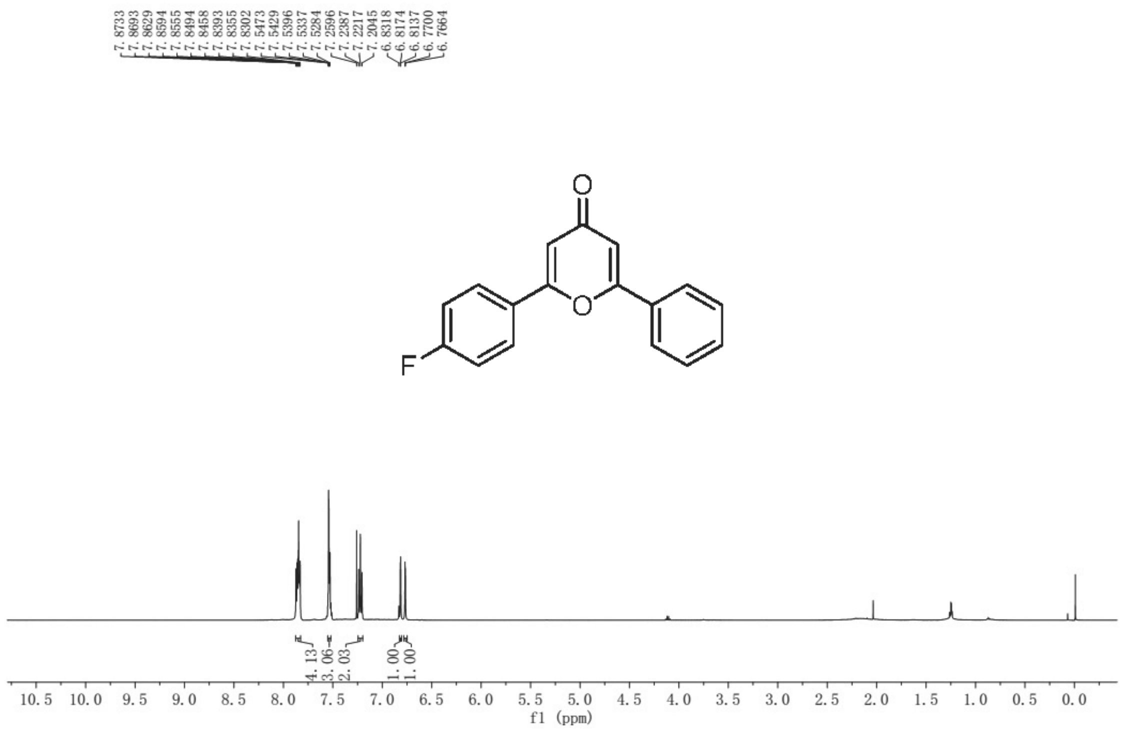

Figure S27. ^{1}H-NMR Spectra of **2n**.

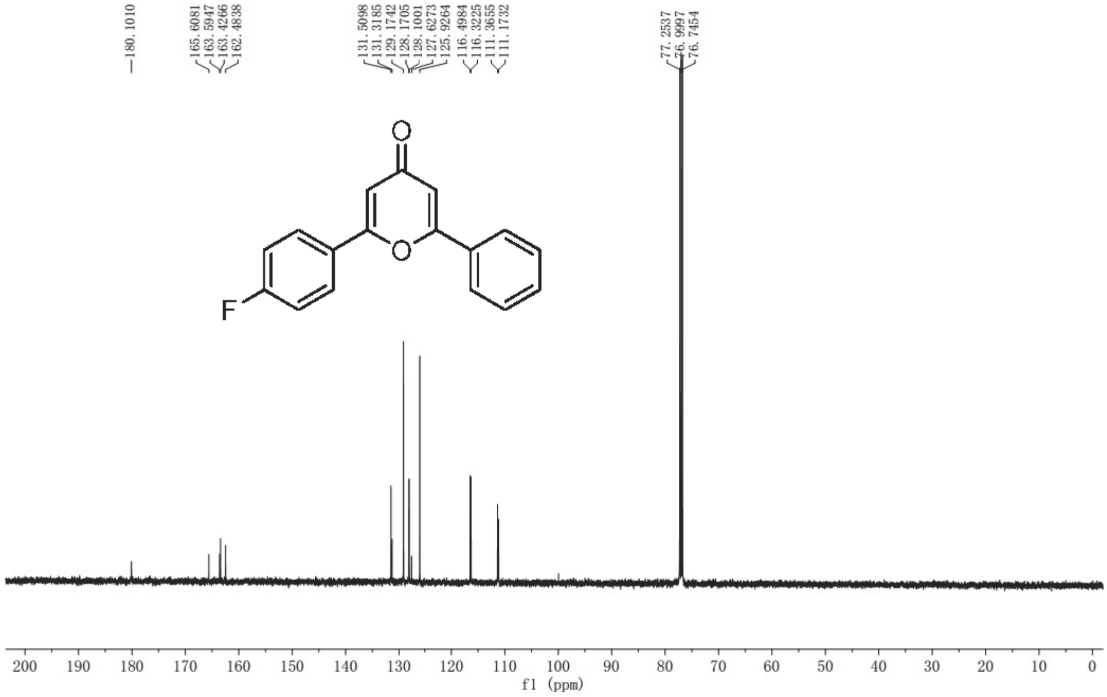

Figure S28. ^{13}C-NMR Spectra of **2n**.

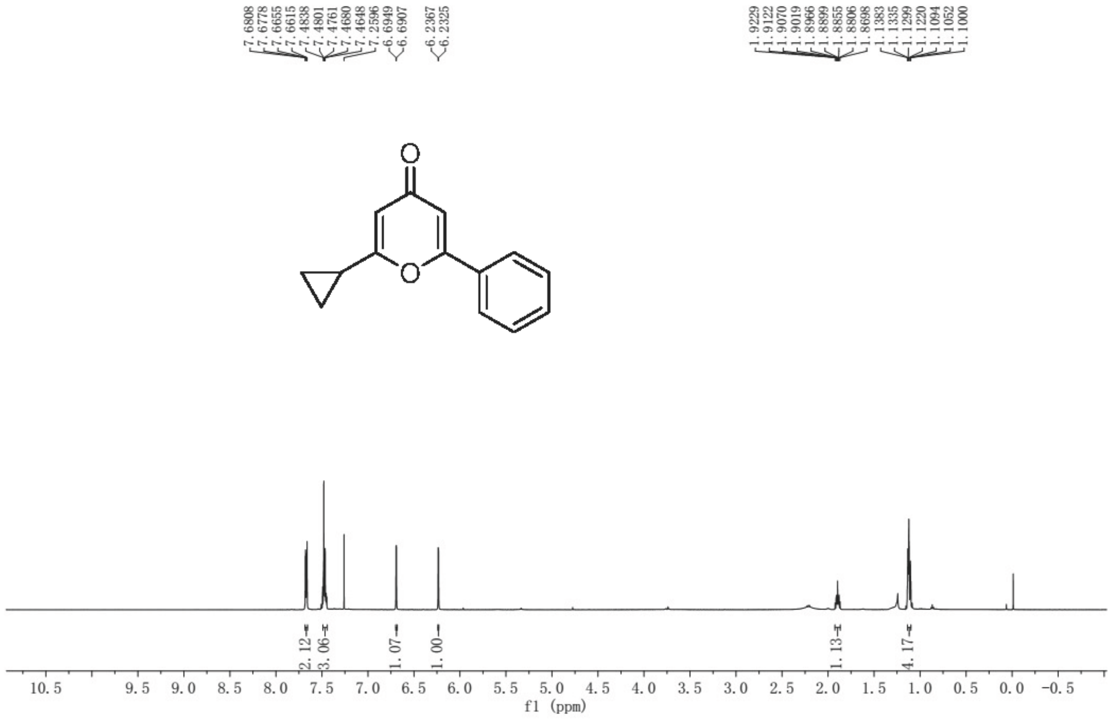

Figure S29. ^{1}H-NMR Spectra of **2o**.

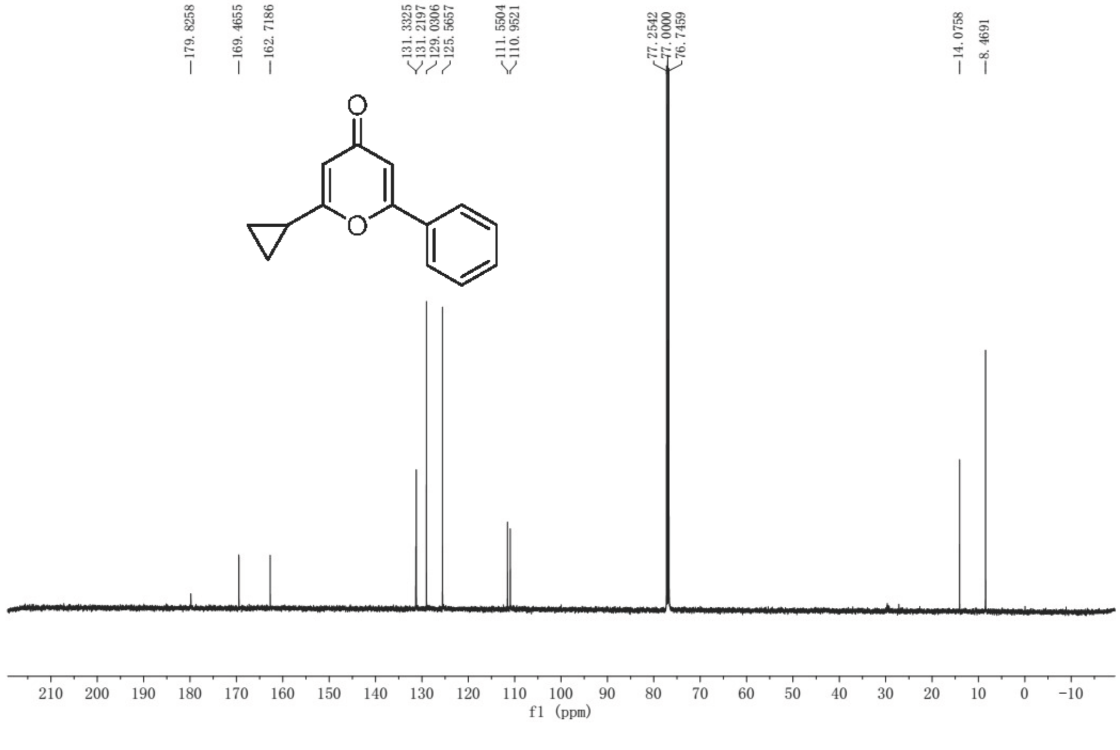

Figure S30. ^{13}C-NMR Spectra of **2o**.

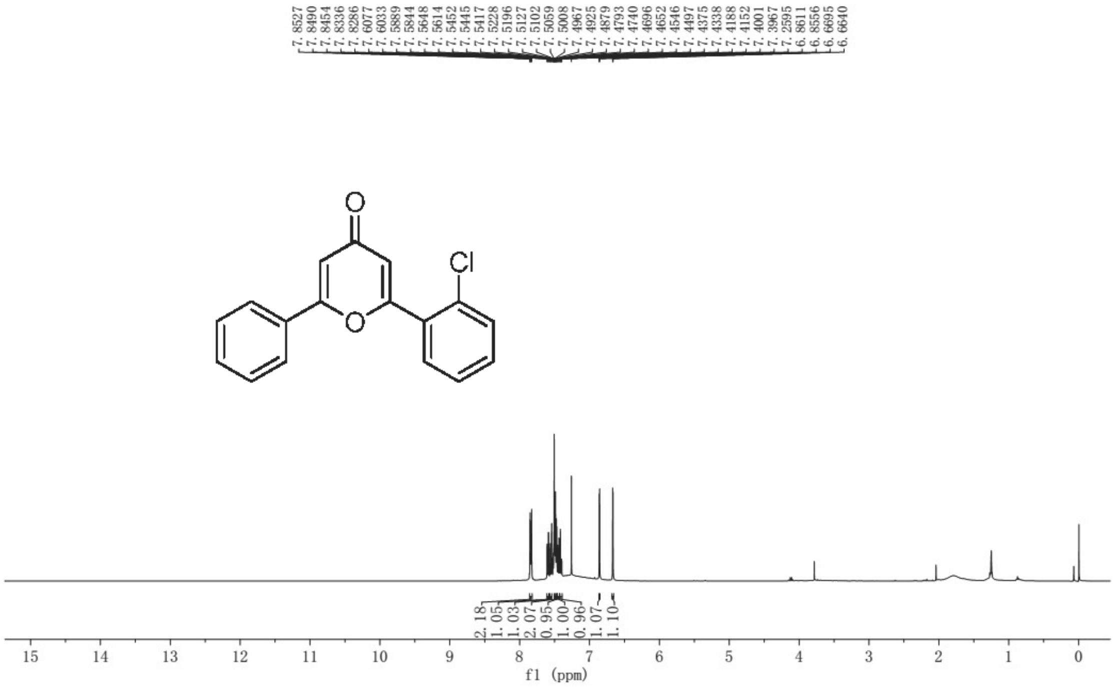

Figure S31. ^{1}H-NMR Spectra of **2p**.

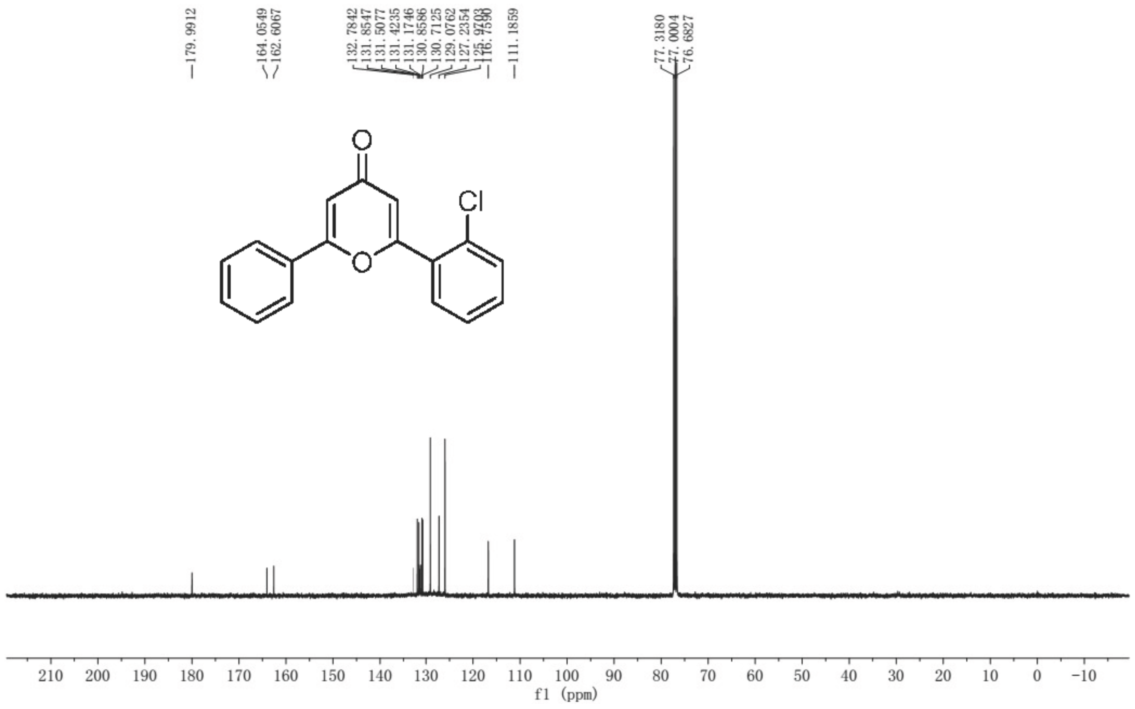

Figure S32. ^{13}C-NMR Spectra of **2p**.

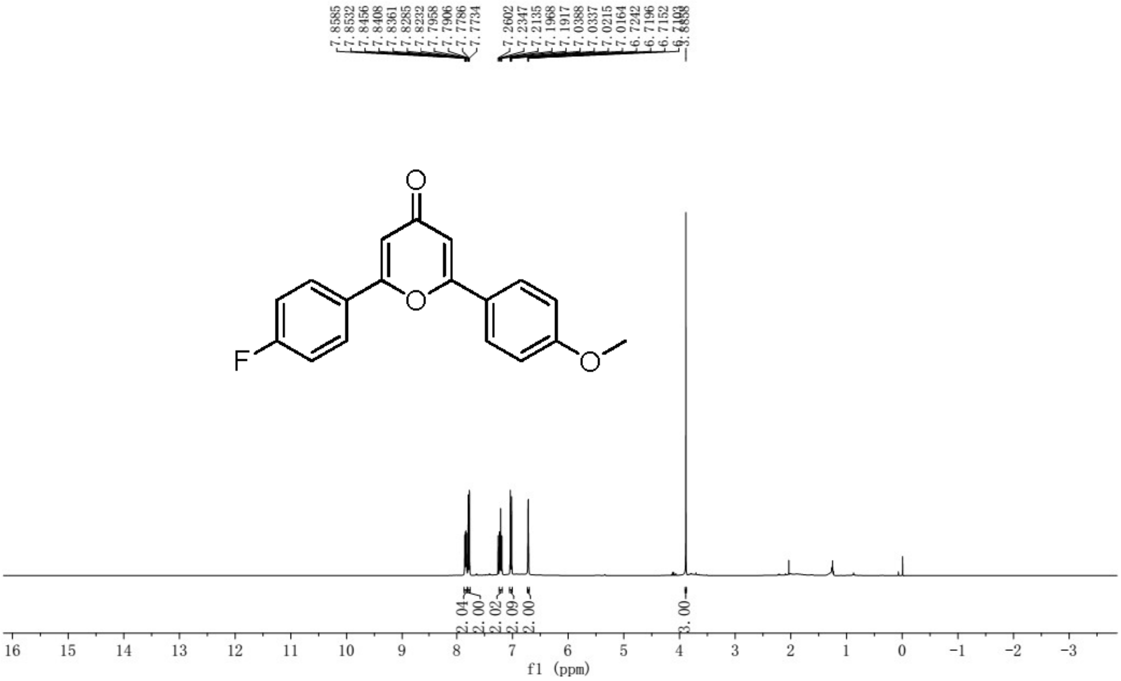

Figure S33. ^{1}H-NMR Spectra of **2q**.

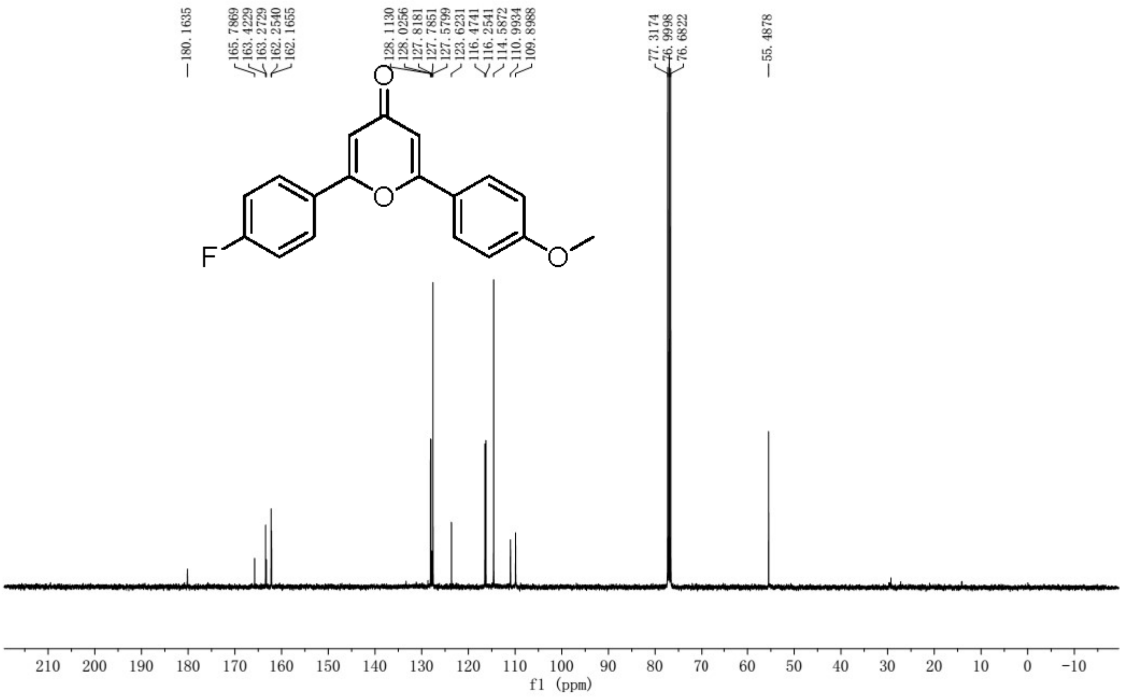

Figure S34. ^{13}C-NMR Spectra of **2q**.

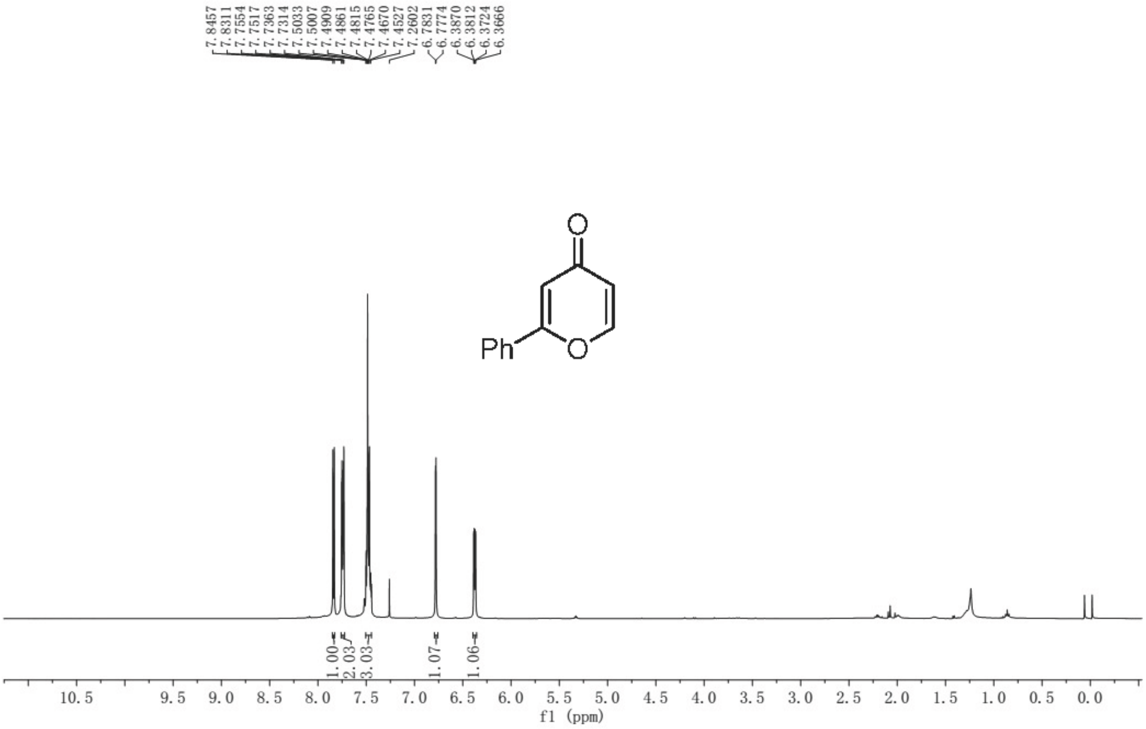

Figure S35. ^{1}H-NMR Spectra of **2r**.

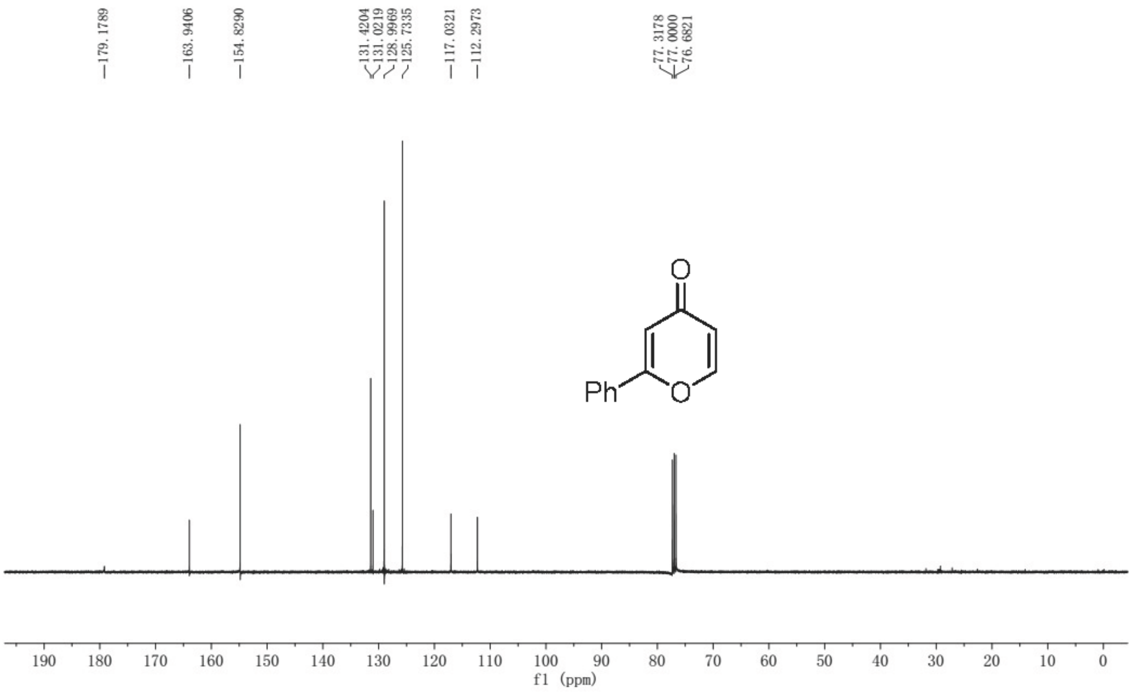

Figure S36. ^{13}C-NMR Spectra of **2r**.

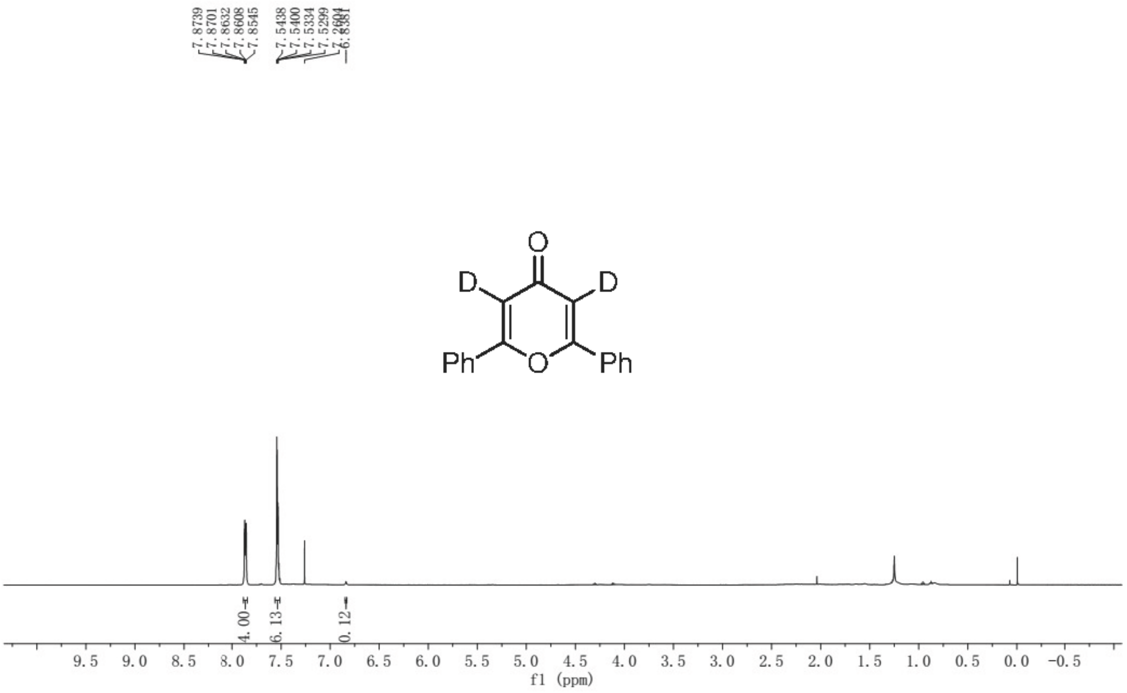

Figure S37. ^{1}H-NMR Spectra of **2a-d**.

2. Copies of HRMS Spectra of Products

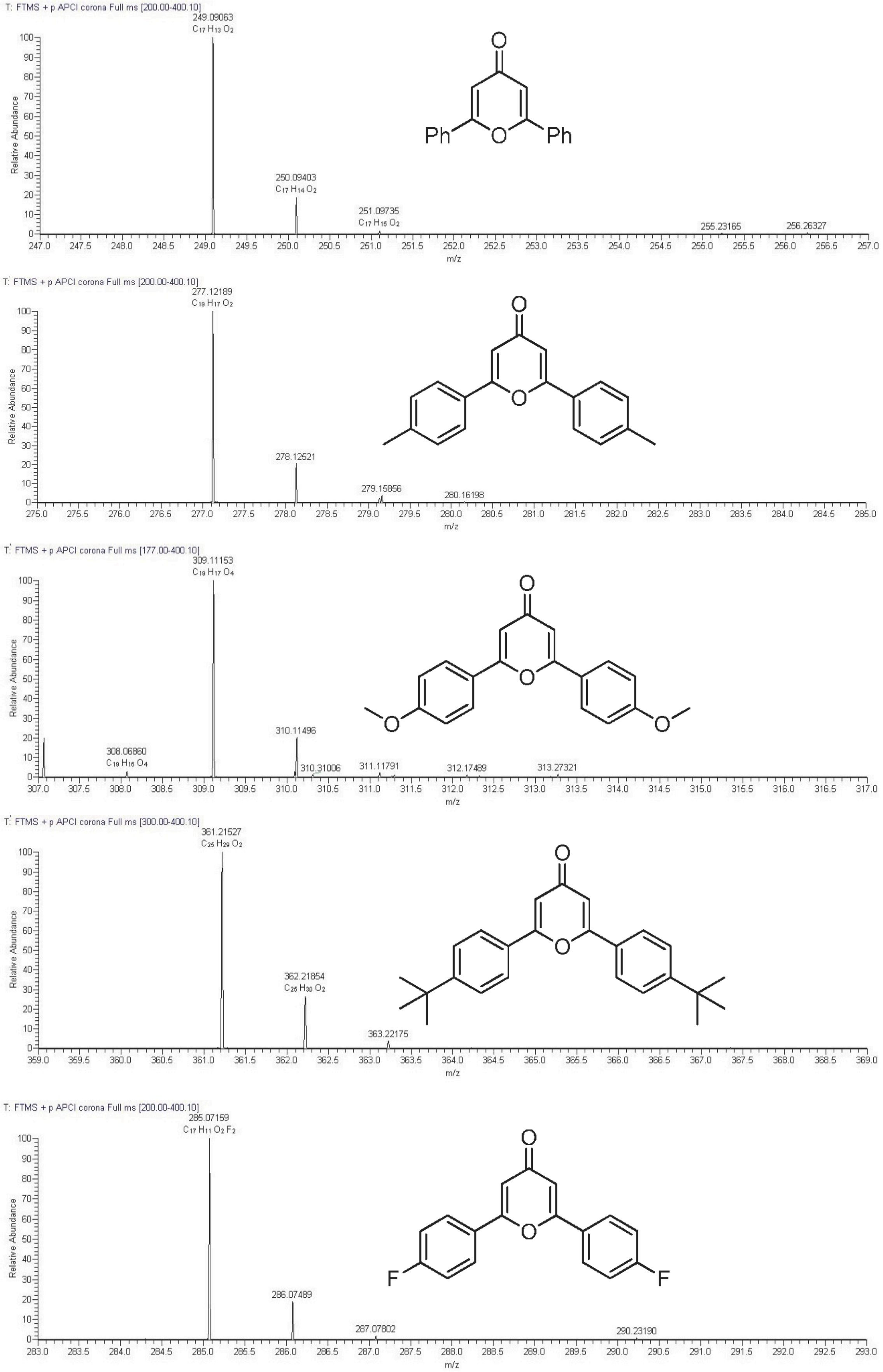

Figure S38. HRMS Spectra of **2a**, **2b**, **2c**, **2d**, **2e**.

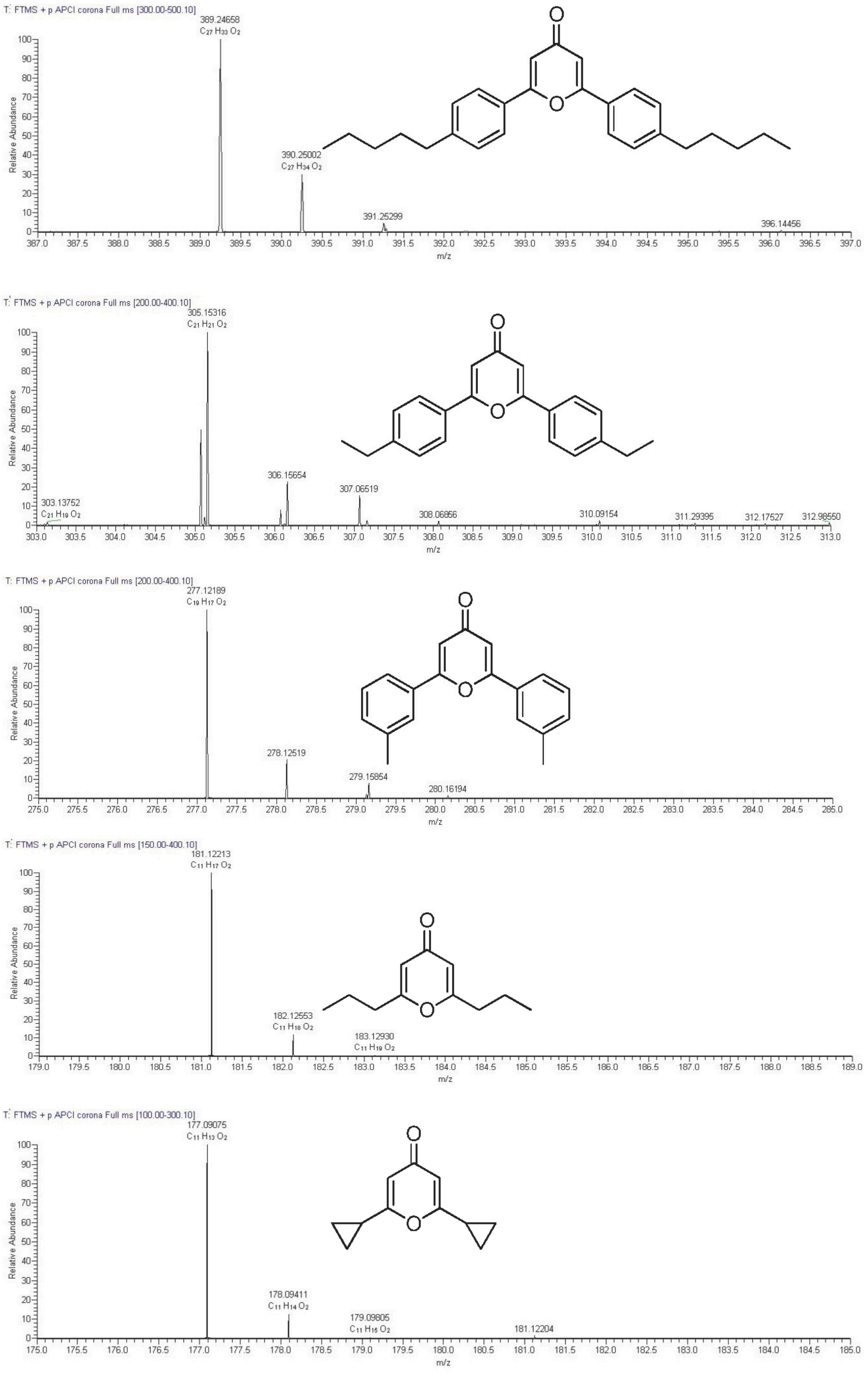

Figure S39. HRMS Spectra of **2f**, **2g**, **2h**, **2i**, **2j**.

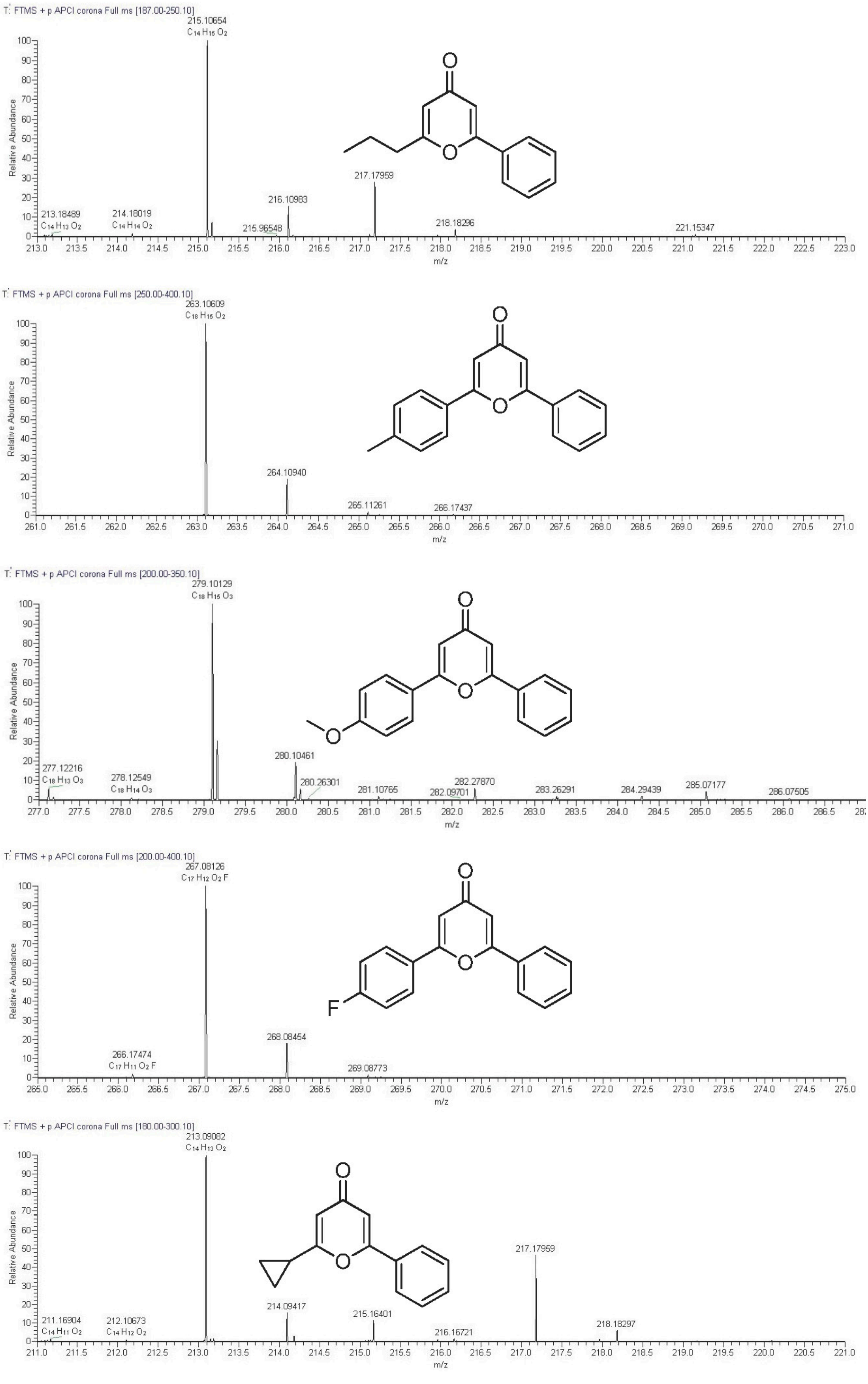

Figure S40. HRMS Spectra of **2k**, **2l**, **2m**, **2n**, **2o**.

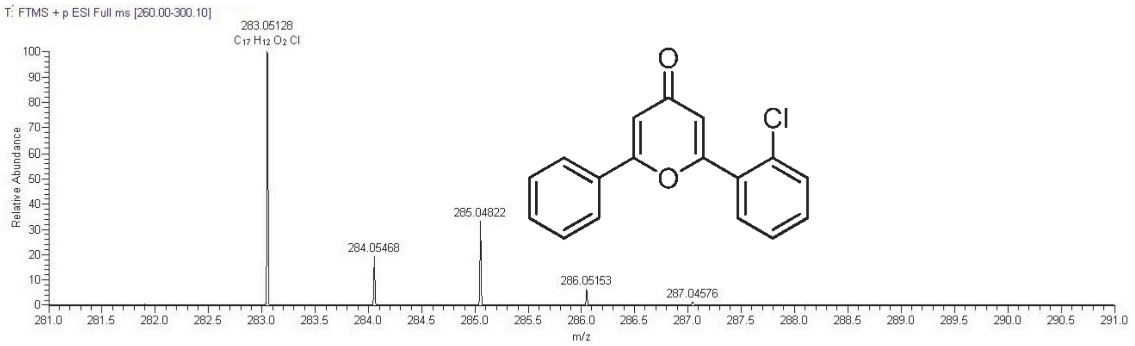

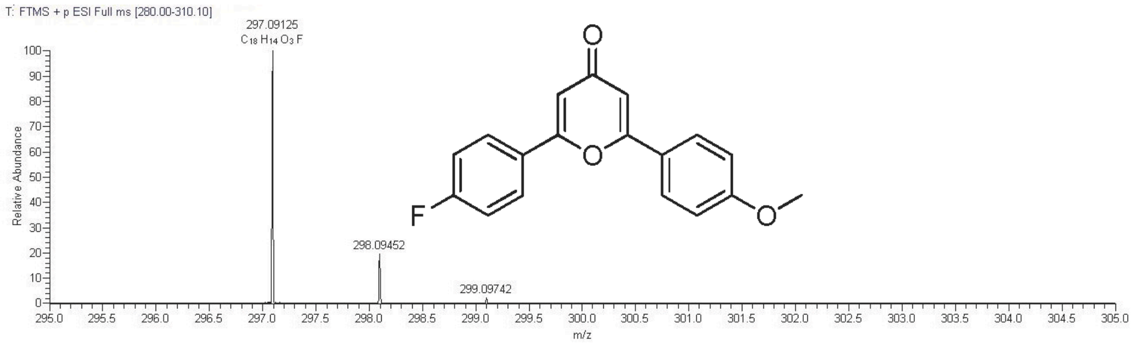

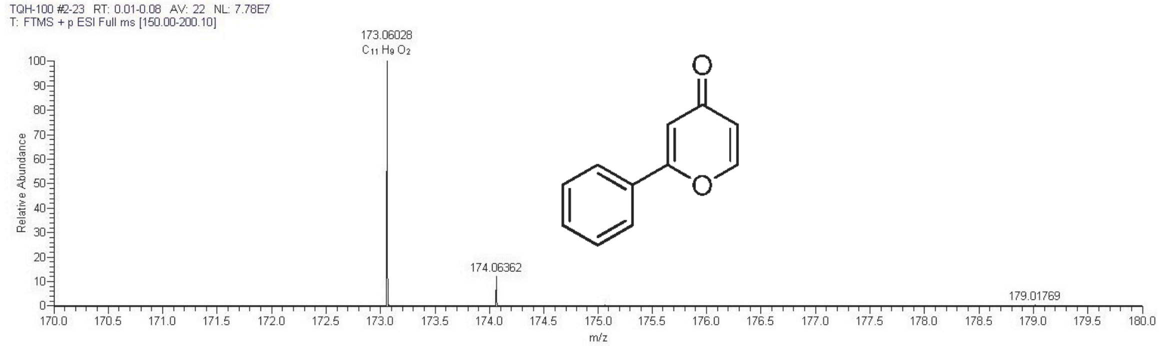

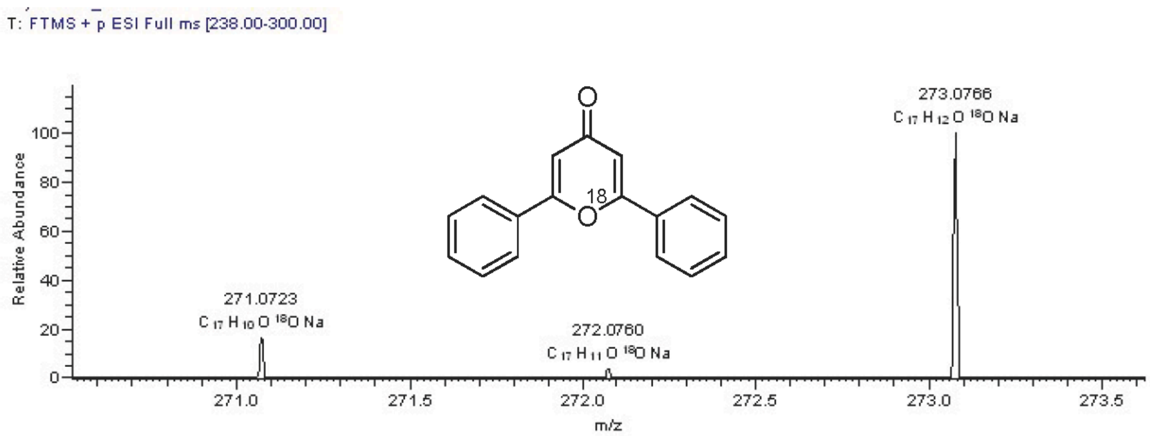

Figure S41. HRMS Spectra of **2p**, **2q**, **2r**, **O[18]-2a**.
